# Predictors of human-infective RNA virus discovery in the United States, China, and Africa, an ecological study

**DOI:** 10.7554/eLife.72123

**Published:** 2022-06-06

**Authors:** Feifei Zhang, Margo Chase-Topping, Chuan-Guo Guo, Mark EJ Woolhouse

**Affiliations:** 1 Usher Institute, University of Edinburgh Edinburgh United Kingdom; 2 Roslin Institute and Royal (Dick) School of Veterinary Studies, University of Edinburgh Edinburgh United Kingdom; 3 Department of Medicine, Li Ka Shing Faculty of Medicine, University of Hong Kong Hong Kong China; Mahidol University Thailand; Pennsylvania State University United States

**Keywords:** emerging virus, risk factor, machine learning, Viruses

## Abstract

**Background:**

The variation in the pathogen type as well as the spatial heterogeneity of predictors make the generality of any associations with pathogen discovery debatable. Our previous work confirmed that the association of a group of predictors differed across different types of RNA viruses, yet there have been no previous comparisons of the specific predictors for RNA virus discovery in different regions. The aim of the current study was to close the gap by investigating whether predictors of discovery rates within three regions—the United States, China, and Africa—differ from one another and from those at the global level.

**Methods:**

Based on a comprehensive list of human-infective RNA viruses, we collated published data on first discovery of each species in each region. We used a Poisson boosted regression tree (BRT) model to examine the relationship between virus discovery and 33 predictors representing climate, socio-economics, land use, and biodiversity across each region separately. The discovery probability in three regions in 2010–2019 was mapped using the fitted models and historical predictors.

**Results:**

The numbers of human-infective virus species discovered in the United States, China, and Africa up to 2019 were 95, 80, and 107 respectively, with China lagging behind the other two regions. In each region, discoveries were clustered in hotspots. BRT modelling suggested that in all three regions RNA virus discovery was better predicted by land use and socio-economic variables than climatic variables and biodiversity, although the relative importance of these predictors varied by region. Map of virus discovery probability in 2010–2019 indicated several new hotspots outside historical high-risk areas. Most new virus species since 2010 in each region (6/6 in the United States, 19/19 in China, 12/19 in Africa) were discovered in high-risk areas as predicted by our model.

**Conclusions:**

The drivers of spatiotemporal variation in virus discovery rates vary in different regions of the world. Within regions virus discovery is driven mainly by land-use and socio-economic variables; climate and biodiversity variables are consistently less important predictors than at a global scale. Potential new discovery hotspots in 2010–2019 are identified. Results from the study could guide active surveillance for new human-infective viruses in local high-risk areas.

**Funding:**

FFZ is funded by the Darwin Trust of Edinburgh (https://darwintrust.bio.ed.ac.uk/). MEJW has received funding from the European Union’s Horizon 2020 research and innovation programme under grant agreement No. 874735 (VEO) (https://www.veo-europe.eu/).

## Introduction

RNA viruses are the primary cause for emerging infectious diseases with epidemic potential, given that they have a high rate of evolution and high capacity to adapt to new hosts (Woolhouse et al., 2016). In recent decades, infectious diseases caused by severe acute respiratory syndrome coronavirus (SARS-CoV), Middle East respiratory syndrome coronavirus (MERS-CoV), Bundibugyo Ebola virus and SARS-CoV-2 present major threats to the health and welfare of humans (Albariño et al., 2013; Ksiazek et al., 2003; Mackay and Arden, 2015; World Health Organisation, 2020). Detection of formerly unknown human-infective RNA viruses in the earliest stage after the emergence are essential for controlling the infections they cause. Measures to implement early detection include not only advanced diagnostic techniques (Lipkin and Firth, 2013), but more importantly the idea where to look for them (so-called hotspots) (Morse, 2012).

Socio-economic, environmental, and ecological factors related to both virus natural history and research effort have been found to affect the discovery of emerging RNA viruses (Jones et al., 2008; Morse, 2012; Rosenberg, 2015; Zhang et al., 2020). However, these factors are highly spatially heterogeneous, making the generality of any associations with discovery debatable. For example, the United States, China, and Africa have experienced different rates of socio-economic, environmental, and ecological changes in the last one hundred years. The United States has always had better resources to discover new viruses. For example, the Rockefeller Foundation—a U.S. foundation—supported the discovery of 23 arboviruses in Latin America, Africa, and India in 1951–1969 (Rosenberg et al., 2013). China has seen urban land coverage more than double and GDP per capita increase by seven times since the 1980s (Ritchie, 2018; Roser, 2013). Nine out of 223 human-infective RNA viruses have been originally discovered in China, and all were discovered after 1982 (Zhang et al., 2020). In contrast, effective surveillance is challenging in less developed regions such as large parts of Africa given resource constraints (Petti et al., 2006).

There have been no previous comparisons of the specific predictors for RNA virus discovery in different regions. In this study, we applied a similar methodology from our previous study of global patterns of discovery of human-infective RNA viruses (Zhang et al., 2020) to investigate whether predictors of discovery rates within three regions—the United States, China, and Africa—differ from one another and from those at the global level, using three new virus discovery data sets. We also mapped discovery probability in three regions in 2010–2019 using the fitted models and historical predictors. According to findings from our previous study (Zhang et al., 2020), the main predictors for virus discovery at the global scale were GDP-related. This suggests that the patterns of virus discovery we have identified may have been largely driven by research effort rather than the underlying biology. In this study, by focusing on more restricted and homogenous regions where the research effort is less variable, we expected to identify predictors more associated with virus biology.

## Materials and methods

### Data sets of human-infective RNA viruses in three regions

We performed an ecological study, and the subject of interest is each human-infective RNA virus species. With reference to a full list of human-infective RNA virus species (Zhang et al., 2020), we geocoded the first report of each in humans in the United States, China, and Africa separately. The latest version as of 31 December 2019 included 223 species (Appendix 1—table 1), with *Human torovirus* abolished and a new species—*Heartland banyangvirus*—added by International Committee on Taxonomy of Viruses (ICTV) in 2018 (International Committee on Taxonomy of Viruses, 2018). Data used in this study were not subsets of our previous global analysis; information on discovery locations and discovery dates for each virus species was re-collated for each specific geographical region.

We followed the same search terms, databases searched, and inclusion or exclusion criteria as our global data set for data collection (Woolhouse and Brierley, 2018). In each region, we established whether or not each virus species has been discovered in humans according to peer-reviewed literature. Reference databases included PubMed, Web of Science, Google Scholar, and Scopus. Two Chinese databases [i.e. China National Knowledge Infrastructure (CNKI) and Wanfang Data] were also searched when collecting data for China. Reference lists of relevant studies and reviews were also checked manually to find potential earlier discovery papers. The following key words were used for the retrieval: virus full name or abbreviations or virus synonyms; and human* or person* or case* or patient* or worker* or infection* or disease* or outbreak* or epidemic*; and region name (Chin* or Taiwan or Hong Kong or Macau; United States or US or USA or America*; Africa* or all African country names). Virus synonyms and abbreviations include early names used in the discovery paper and all subtypes provided by the ICTV online report (International Committee on Taxonomy of Viruses, 2018 ). Evidence which met the following criteria from peer-reviewed literatures were included: (a) Diagnostic methods for RNA virus infection in humans were clearly described, through either viral isolation or serological methods; (b) Specific virus species name or subtypes falling under that species were clearly provided; (c) Both natural infection and iatrogenic or occupational infections were accepted. Evidence which met the following criteria were excluded: (a) Uncertain species due to cross-reactivity with related viruses; (b) Diagnostic methods for virus infection were not specified; (c) Description of clinical symptoms or pathogenicity were not considered as human infection of one certain virus species; (d) Report of ‘[virus name]-like’ or ‘potential [virus name] infections’; (e) Intentional infections including experimental inoculation or vitro infections; (f) Non-peer-reviewed literature, including media reports, thesis, or unpublished data. Literature selection was performed by two individuals independently and discrepancies were resolved by discussion with a third individual.

We defined discovery location as where the initial human was exposed to/infected with the virus, as suggested in the first report of human infections from peer-reviewed literature. All locations were geolocated as precisely as possible using methods from our previous paper (Zhang et al., 2020). For each region, a polygon was created for those locations at administrative level 3 (county for the United States; city for China; for Africa, it varies between different countries) and above. Details of data types for virus discovery database in three regions was summarised in Appendix 1—table 2. Although the majority of discovery locations in the United States and Africa involved point data and in China the majority involved polygon data at province level, the average number of grid cells per virus in three regions were similar. A bootstrap resampling procedure was developed for polygon data covering more than one grid cell (details below). Discovery date of human infection was defined as the publication year in the scientific literature.

### Spatial covariates

As for our global analysis (Zhang et al., 2020), a suite of global gridded climatic, socio-economic, land use, and biodiversity variables (n=33) postulated to affect the spatial distribution of RNA virus discovery were compiled, each at a resolution of 0.5°/30" (except university count having a resolution at country level for Africa and at state/province level for the United States and China). Of these, GDP, GDP growth, and university were included to adjust for discovery effort as they could partially explain the infrastructure and technology that are available for virus research (Zhang et al., 2020). We reviewed and tested previous strategies researchers have used to adjust for discovery bias, including frequency of the country listed as the address for authors in scientific papers and frequency of publications for each pathogen from scientific databases (Jones et al., 2008; Olival et al., 2017) but the results were not encouraging as the frequency of published papers from virus-related scientific journals is weakly linked to the published count of novel human-infective RNA virus (Appendix2, Appendix 3—figure 1).

Data for the United States, China, and Africa were extracted by restricting the coordinates within each region. The definition, original resolution, and source of each variable were the same as our previous paper (Zhang et al., 2020). All predictors were aggregated from their original spatial resolution to 1°×1° resolution; data for climatic variables, population, GDP, and land use data without full temporal coverage were extrapolated back to 1901; both following methods from our previous paper (Zhang et al., 2020).

### Boosted regression trees modelling

We used a Poisson boosted regression trees (BRT) model to examine the relationship between discovery of RNA virus and 33 predictors for each 1° resolution of grid cell across each region separately, following codes from our previous study (Zhang et al., 2020) and one previous paper (Allen et al., 2017). As a tree-based machine learning method, the BRT model can automatically capture complex relationships and interactions between variables, and also can well account for spatial autocorrelation within the data (Crase et al., 2012). We compared Moran’s I values of the raw virus data and the model residuals to estimate the ability of the BRT model to account for spatial autocorrelation (Cliff and Ord, 1981). In order to minimise the effect of spatial uncertainty of virus discovery data, we performed 1000 times bootstrap resampling for those discovery locations reported as polygons. We assumed each grid cell in the polygon has the equal chance to be selected, and for each virus record we selected one grid cell randomly from the polygon for each subsample. A ratio of 1:2 for presence to absence constituted each subsample, that is, for each grid cell with virus discovery, two grid cells with no discovery were randomly selected from ‘virus discovery free’ areas at all time points within the region. Take the United States as an example, each subsample included 95 grid cells with virus discovery and 190 with no virus discovery. We then matched the virus data with all predictors by geographical coordinates and decade (using the nearest decade for time-varying predictors). We assumed that the virus count in any given grid cell in each decade followed a Poisson distribution, and we calculated the virus discovery count in each grid cell by decade as the response variable.We also performed further sensitivity analyses by (i) matching virus discovery data and time-varying covariate data by year and (ii) testing for lag effects by matching virus discovery at year t and predictors at t-1 to t-5 year (Appendix4).

All BRT models were fitted in R v. 3.6.3, using packages dismo and gbm. BRT models require the user to balance three parameters including tree complexity, learning rate, and bag fraction. Tree complexity reflects the order of interaction in a tree; learning rate shrinks the contribution of each tree to the growing model; bag fraction specifies the proportion of data drawn from the full training data at each step. We set these parameters as recommended from Elith et al., 2008, and make sure each resampling model contained at least 1000 trees. BRT models identified the final optimal number of trees in each model using a 10-fold cross validation stagewise function (Elith et al., 2008). The three parameter values of the optimal model as well as the mean optimal number of trees across 1000 replicate models for all three regions were summarised in Appendix 1—table 3.

By fitting 1000 replicate BRT models, the relative contribution plots and partial dependence plots with 95% quantiles were plotted. We defined variables with a relative contribution greater than the mean (3.03%) as influential predictors in all three regions (Shearer et al., 2018). The partial dependence plots depict the influence of each variable on the response while controlling for the average effects of all the other variables in the model. The map of virus discovery probability across each region in 2010–2019 was derived from the means of the predictions of 1000 replicate models, using values of the 33 predictors in 2015. In order to show discovery hotspots, we converted the prediction map of virus count to a map of probability.

Two statistics were calculated to evaluate the model’s predictive performance: (a) the deviance of the bootstrap model (Elith et al., 2008), (b) intraclass correlation coefficient (ICC) calculated from 50 rounds of 10-fold cross-validation, by following methods from our previous paper (Zhang et al., 2020). For the 10-fold cross-validation, we selected 50 data sets randomly from the 1000 bootstrapped subsamples. We took the first data set and partitioned into 10 subsets. For each round of 10-fold cross-validation, the unique combinations of nine subsets constituted the training sets and were used to fit models, and the remaining one was used as a test set to evaluate the predictive performance of the model. We repeated the same process as above for the remaining 49 data sets. One intraclass correlation coefficient (ICC) was calculated from each round of validation and the median with 95% quantiles across all 50 rounds was calculated. The ICC varies between 0 and 1, with an ICC of less than 0.40 representing a poor model, 0.40–0.59 representing a fair model, 0.60–0.74 representing a good model, and 0.75–1 representing an excellent model (Cicchetti, 1994).

Exploratory subgroup analyses distinguishing viruses firstly discovered in regions and those that had been discovered elsewhere in the world were performed. We used the same BRT modelling approach as we described above, and relative contribution of each predictor was calculated for each subgroup. We were unable to perform subgroup analysis for China because only nine human-infective RNA viruses have been firstly discovered in it, and the BRT model cannot be fitted to a sample as small as 9.

R software, version 3.6.3 (R Foundation for Statistical Computing, Vienna, Austria) was used for all statistical analyses. All maps were visualised by using ArcGIS Desktop 10.5.1 (Environmental Systems Research Institute).

## Results

**Figure 1.**
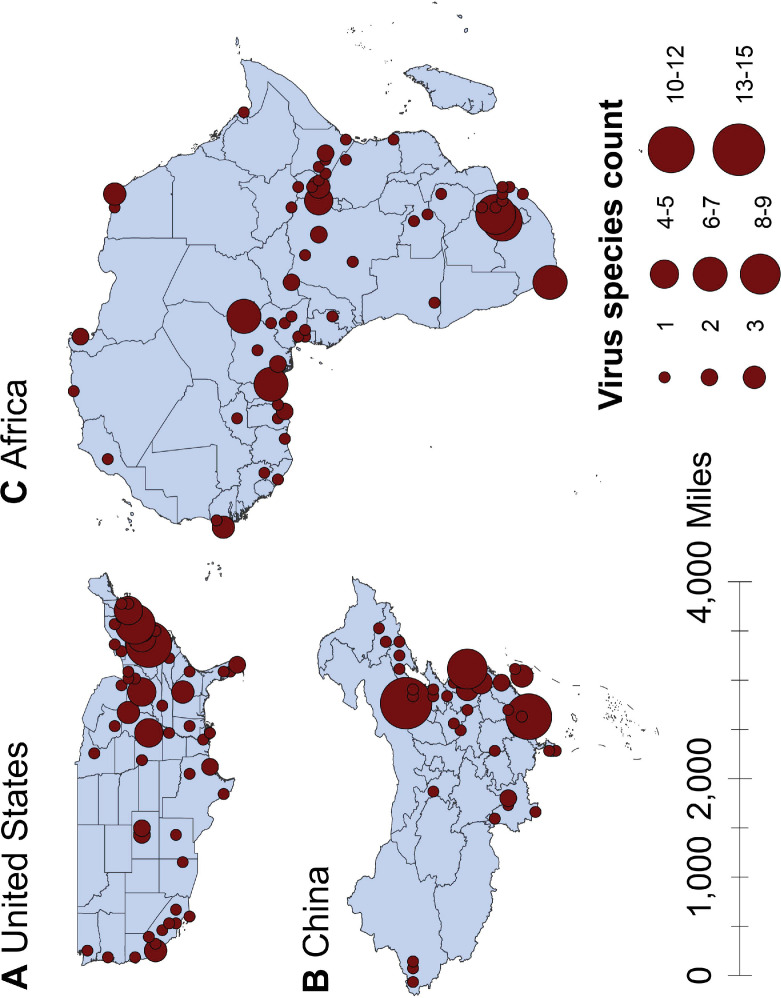
Spatial distribution of human-infective RNA virus discovery in three regions, 1901–2019. (**A**) United States. (**B**) China. (**C**) Africa. Red dots represent discovery points or centroids of polygons, with the size representing the cumulative virus species count.

**Figure 2.**
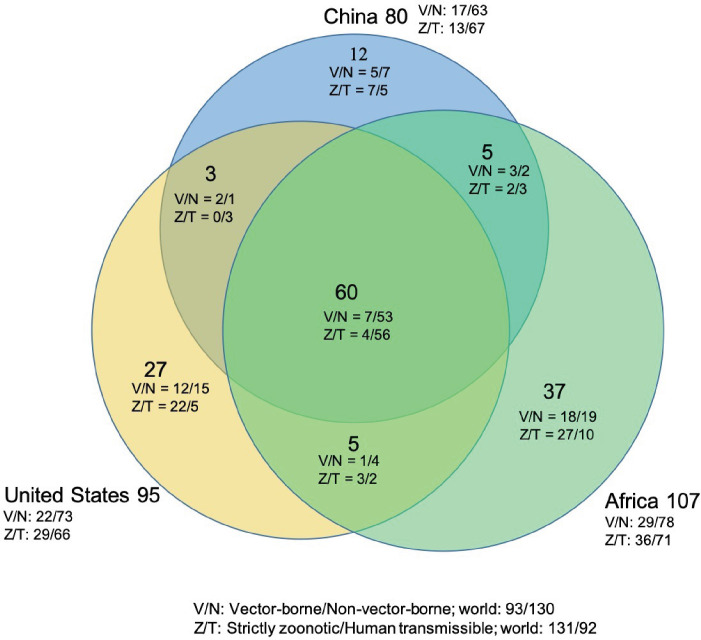
Shared human-infective RNA virus species count in three regions. Under/By the species count the ratios of vector-borne (**V**) to non-vector-borne (**N**) viruses and strictly zoonotic (**Z**) to human transmissible (**T**) viruses were shown.

The numbers of human-infective virus species discovered in the United States, China, and Africa up to October 2019 were 95, 80, and 107, respectively (Appendix 1—table 1). Most first discoveries have been in eastern United States (especially in areas around Maryland, Washington, D.C., and New York), eastern China (developed cities including Beijing, Hong Kong, Shanghai, and Guangzhou), and southern and central Africa (Pretoria and Johannesburg, South Africa; Borno State and Ibadan, Nigeria) (Figure 1). A total of 60 virus species were previously reported in all three regions, and 27, 12, 37 species were only found in the United States, China, and Africa, respectively (Figure 2). In all three regions, smaller proportions of viruses were vector-borne [United States: 23.2% (22/95); China: 21.3% (17/80); Africa: 27.1% (29/107)] and strictly zoonotic [United States: 30.5% (29/95); China: 16.3% (13/80); Africa: 33.6% (36/107)], compared to large proportions for both virus types at the global scale [vector-borne: 41.7% (93/223) and strictly zoonotic: 58.7% (131/223)] (Figure 2). The 60 shared species were also disproportionally vector-borne [11.7% (7/60)] and strictly zoonotic [7% (4/60), Figure 2].

**Figure 3.**
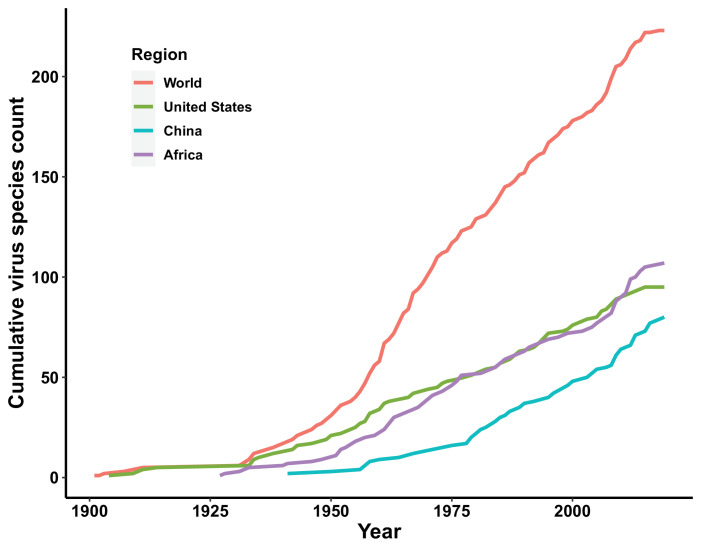
Discovery curve of human-infective RNA virus species in three regions and the world.

The discovery curves for the United States and Africa have seen a broadly similar pattern, with China lagging behind these two regions (Figure 3). The median time lag between the original discovery year of each virus in the world and the discovery year of each virus in each region was 0 [interquartile range (IQR): 2.5], 12 (IQR: 29.5), and 2 (IQR: 10.5) years in the United States, China, and Africa, respectively (Appendix 3—figure 2). In China, the time lag was noticeably shorter for viruses discovered after 1975 [before 1975: a median lag of 30.5 (IQR: 30.5) years; after 1975: 2.5 (IQR: 7) years, p value of Wilcoxon rank sum test < 0.001].

**Figure 4.**
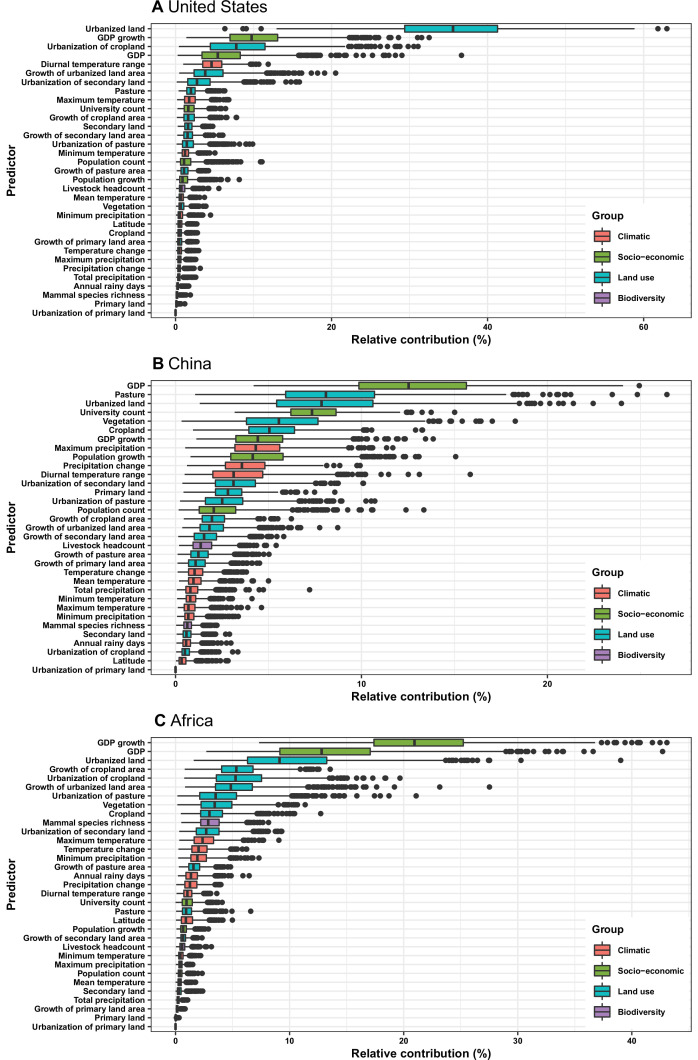
Relative contribution of predictors to human-infective RNA virus discovery in three regions. (**A**) United States. (**B**) China. (**C**) Africa. The boxplots show the median (black bar) and interquartile range (box) of the relative contribution across 1000 replicate boosted regression tree models, with whiskers indicating minimum and maximum and black dots indicating outliers.

In the United States, six variables including three predictors related to land use [urbanized land: relative contribution of 35.8%, urbanization of cropland (i.e. the percentage of land area change from cropland to urban land): 8.0%, growth of urbanized land: 4.1%], two socio-economic variables (GDP growth: 10.0%; GDP: 5.7%), and one climatic variable (diurnal temperature change: 4.9%) were identified as important predictors for discriminating between locations with and without virus discovery (Figure 4A). The partial dependence plots shown in Appendix 3—figure 3 suggested non-linear relationships between the probability of virus discovery and most predictors. All important predictors presented a positive trend over narrow ranges at lower values.

In China, twelve variables including four socio-economic variables (GDP: 12.7%, university count: 7.5%, GDP growth: 4.6%, population growth: 4.4%), five predictors involving land use [pasture: 8.3%, urbanized land: 8.1%, vegetation: 5.8%, cropland: 5.3%, urbanization of secondary land (the percentage of land area change from secondary land to urban land; secondary land is natural vegetation that is recovering from previous human disturbance): 3.3%], and three climatic variables (maximum precipitation: 4.5%, precipitation change: 3.8%, diurnal temperature range: 3.3%) were identified as important predictors for discriminating between locations with and without virus discovery (Figure 4B). GDP, urbanized land, university count, vegetation, GDP growth, maximum precipitation, population growth, and urbanization of secondary land presented a positive trend over narrow ranges at lower levels; pasture, cropland, precipitation change, and diurnal temperature range had non-monotonic/ negative impacts, with highest risks at lower values (Appendix 3—figure 4).

In Africa, ten variables including two socio-economic variables (GDP growth: 21.2%, GDP: 13.0%), seven predictors related to land use (urbanized land: 9.4%, growth of cropland area: 5.6%, urbanization of cropland: 5.5%, growth of urbanized land: 5.1%, urbanization of pasture: 3.8%, vegetation, 3.7%, cropland: 3.2%), and one biodiversity variable (mammal species richness: 3.1%) were identified as important predictors for discriminating between locations with and without virus discovery (Figure 4C). All important predictors presented a positive trend over narrow ranges at lower positive values, except mammal species over a large range (Appendix 3—figure 5).

Our BRT models reduced Moran’s I value below 0.15 in all three regions (Appendix 3—figure 6), suggesting that BRT models with 33 predictors have adequately accounted for spatial autocorrelations in the raw virus data in all three regions. The model validation statistics for each region are shown in Appendix 1—table 4. Combining these measures, our BRT model predictions range from fair to good (Cicchetti, 1994). In our sensitivity analyses based on data matched by year (Appendix 3—figure 7) and 1–5 year lag (results of 1 year lag shown in Appendix 3—figure 8), though there were several changes of relative contribution, the top predictors were broadly consistent with our main model based on data matched by decade (Figure 4).

**Figure 5.**
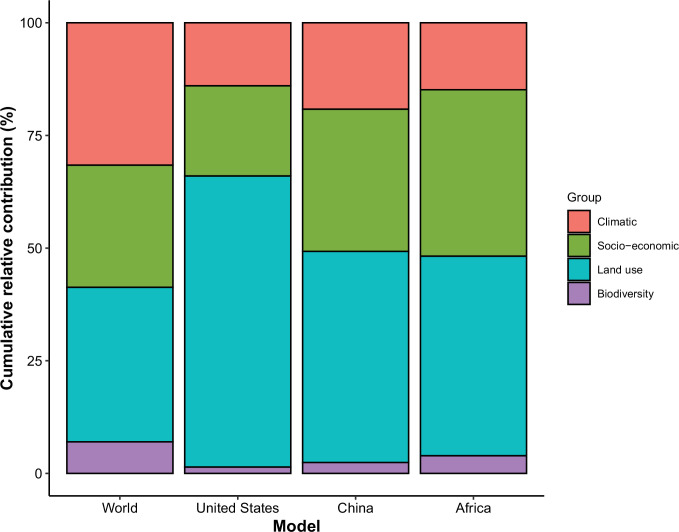
Cumulative relative contribution of predictors to human-infective RNA virus discovery by group in each model of different regions. The relative contributions of all explanatory factors sum to 100% in each model, and each colour represents the cumulative relative contribution of all explanatory factors within each group.

In comparison with the whole world, human-infective RNA virus discovery was more associated with land use and socio-economic variables than climatic variables and biodiversity in all three regions (Figure 5). The comparison of four groups of predictors between three regions showed that: the greatest contribution of climatic variables to the discovery of human-infective RNA viruses was in China; the greatest contribution of land use was in the United States; the greatest contribution of socio-economic variables and biodiversity was in Africa and least in the United States.

**Figure 6.**
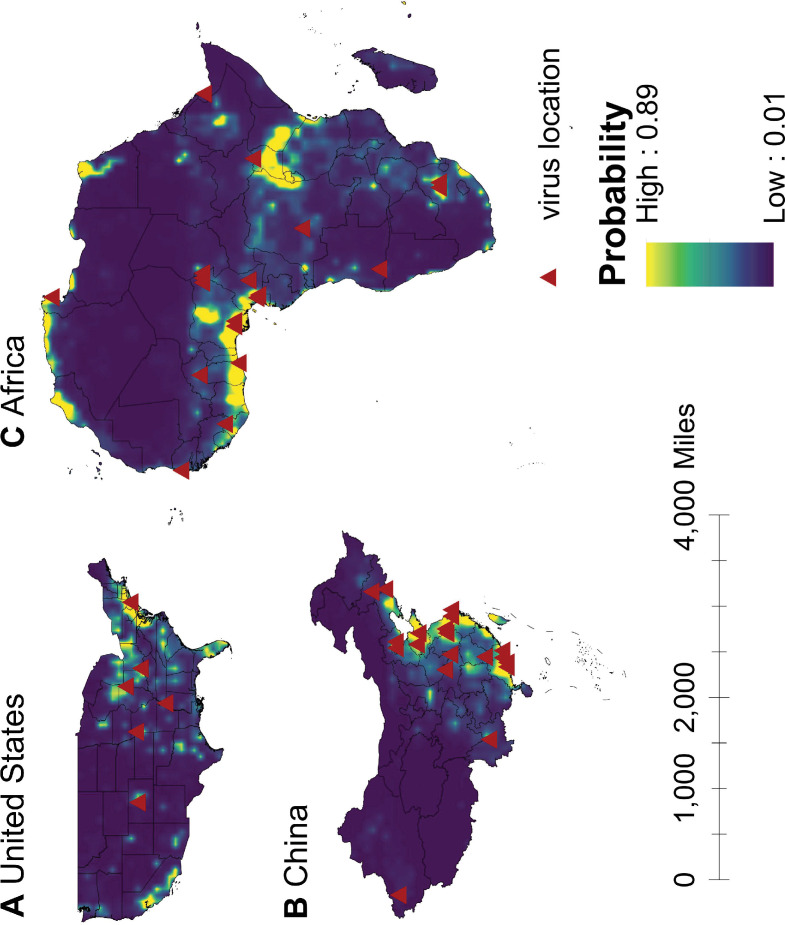
Predicted probability of human-infective RNA virus discovery in three regions in 2010–2019. (A) United States. (B) China. (C) Africa. The triangles represented the actual discovery sites from 2010 to 2019, and the background colour represented the predicted discovery probability.

We mapped human-infective RNA virus discovery probability in 2010–2019 for the three regions, based on the fitted BRT models and values of all 33 predictors in 2015 (Appendix 3—figure 9 to Appendix 3—figure 11). Outside contemporary risk areas where human-infective RNA viruses were previously discovered in the United States (Figure 1A), we predicted high probabilities of virus discovery across southern Michigan, central-Northern Carolina, central Oklahoma, southern Nevada, and north-eastern Utah (Figure 6A). Outside contemporary risk areas where human-infective RNA viruses were previously discovered in China (Figure 1B), we predicted high probabilities of virus discovery across other eastern China area as well as two western areas including south-central Shaanxi and north-eastern Sichuan (Figure 6B). Outside contemporary risk areas where human-infective RNA viruses were previously discovered in Africa (Figure 1C), we predicted high probabilities of virus discovery across northern Morocco, northern Algeria, northern Libya, south-eastern Sudan, central Ethiopia and western Democratic Republic of the Congo (Figure 6C). Most new virus species since 2010 in each region (6/6 in the United States, 19/19 in China, 12/19 in Africa) were discovered in high-risk areas (85% percentiles of predicted probability across each region) as predicted by our model. Of all the 37 (United States: 6; China: 19; Africa: 12) viruses discovered in high-risk areas in 2010–2019, 13 (United States: 2; China: 7; Africa: 4) viruses were discovered at the potential new hotspots where there have not been any virus discoveries before 2010.

Based on our subgroup analysis distinguishing viruses firstly discovered in regions and those that had been discovered elsewhere in the world, discoveries of human-infective RNA viruses first discovered from either United States or Africa were better predicted by climatic and biodiversity variables, while discoveries of viruses that had been discovered from elsewhere in the world were better predicted by socio-economic variables (Appendix 3—figure 12).

## Discussion

To our knowledge, this analysis represents the first investigation of human-infective RNA virus discovery in three large regions of the world which have experienced distinct socio-economic, ecological and environmental changes over the last 100 years. In total, 95 human-infective RNA virus species had been found in the United States; 80 in China; 107 in Africa. The discovery maps of human-infective RNA virus in the three regions indicated areas with historically high discovery counts: eastern and western United States, eastern China, and central and southern Africa. BRT modelling suggested that the relative contribution of 33 predictors to human-infective RNA virus discovery varied across three regions, though climatic and biodiversity variables were consistently less important in all three regions than at a global scale. We mapped the probability of human-infective RNA virus discovery in 2010–2019 which would continue to be high in historical hotspots but, in addition, we identified several new hotspots in central-eastern and southwestern United States, eastern and western China, and northern Africa. These results offer a tool for public health practitioners and policymakers to better understand local patterns of virus discovery and to invest efficiently in surveillance systems at the local level.

In recent decades, factors that drive pathogen discovery have been comprehensively studied, e.g., (Morse, 2012). In general, evidence has come from three forms of analyses: analysis of single emergence event such as SARS, AIDS, and Ebola (Parrish et al., 2008), quantifying the spillover (or host switching/cross-host transmission) risk using traits of both hosts and viruses (Kreuder Johnson et al., 2015; Olival et al., 2017; Pulliam and Dushoff, 2009), and record of first emergence/discovery event in humans globally over time (Allen et al., 2017; Jones et al., 2008; Zhang et al., 2020). Of these, the latter form of analyses have linked the distribution of emerging infectious diseases across the globe to ecological, environmental, and socio-economic factors, predicted the high-risk areas for discovery of emerging zoonoses, and helped identify priority regions for investment in surveillance systems for new human viruses (Allen et al., 2017; Jones et al., 2008; Zhang et al., 2020). In addition to these analyses, our current regional analyses identified more precise hotspots for virus discovery in three large regions of the world. Because zoonotic viruses are responsible for most historical endemics and epidemic diseases, several projects such as the Global Virome project (GVP), the PREDICT project, and the Vietnam Initiative on Zoonotic Infections (VIZIONS) were launched to construct a comprehensive data set of unknown viruses with epidemic potential from specific animals likely to harbour high-risk viruses, humans having a high contacting rate with animals, and animal-human interfaces with high spill-over probability (Carroll et al., 2018; Morse, 2012; Rabaa, 2015). These hotspots analyses indicate priority regions for surveillance for new viruses for these projects.

In all three regions, GDP and/or GDP growth were identified as important predictors for virus discovery. This is consistent with our previous analysis that GDP and GDP growth play a major role in discovering viruses (Zhang et al., 2020). In general, sufficient economic, human and material resources, the availability of advanced infrastructure and technology, and greater research capabilities in the relative higher income areas enable the virus discovery (Rosenberg et al., 2013). That this effect applied both within one continent and within single countries such as the United States and China suggested that most virus discoveries were likely passive, that is, the viruses were detected when they arrived in a location with the resources to detect them. This is plausible because in all regions in our study, human-transmissible viruses accounted for the larger proportion, and our previous analysis suggested richer areas were more likely to first capture transmissible viruses (e.g. Influenza virus, Rhinovirus, Rabies lyssavirus, Measles morbillivirus, Mumps orthorubulavirus, Rubella virus, and Norwalk virus) capable of spreading to multiple areas (Zhang et al., 2020). Temporally, in China the rate of discovery increased after economic growth accelerated in the 1980s (Figure 3). We note in publications describing first virus discoveries that most historical virus discoveries in Africa received support from the United States and Europe, and this may explain why Africa saw an increased number of virus discoveries after 1950—30 years earlier than China (Figure 3). Notably, in contrast to Africa, university count was found to be associated with virus discovery in China, suggesting virus discovery likely being a significant area of research in Chinese universities. Our model also suggested the overall socio-economic factors contributed less in the United States than other two regions. The possible explanation is that the socio-economic level across the whole United States is relatively high and homogenous.

Predictors other than GDP and university count are likely to be linked to virus natural history. In all three regions, the area of urban land and further urbanization made great contribution to virus discovery. This reinforced previous studies that urbanization was linked to the detection of new human pathogens through the denser urban population, increased human-wildlife contact rate, spill-over of human infection from enzootic cycle, and the contamination of the urban environment with microbial agents (Hassell et al., 2017; Olival et al., 2017; Weaver, 2013). In the United States, land use contributed more to virus discovery than in other regions—urbanized land, urbanization of cropland, and growth of urbanized land alone had a relative contribution of 47.9%. It is possible that land use change in the US is driving both the emergence of novel viruses and their discovery, as has been suggested for Heartland virus (Mansfield et al., 2017; Savage et al., 2013) and several hantaviruses (Hassell et al., 2017).

Climate had less influence on human-infective RNA virus discovery in all three regions in comparison to other predictors, in contrast to virus discovery at a global scale (Zhang et al., 2020). The underlying reason may be that the proportion of vector-borne viruses—whose distribution and abundance is strongly associated with the impact of climate on vector populations (Li et al., 2014)—in all three regions (United States: 23.2%; China: 21.3%; Africa: 27.1%) were less than that in the world (41.7%) (Figure 3). Vector-borne viruses tend to have more restricted global ranges, so are less likely to appear in a study of any one region (Zhang et al., 2020).

In addition, a relatively smaller proportion of strictly zoonotic viruses in three regions (United States: 30.5%; China: 16.3%; Africa: 33.6%) than that in the world (58.7%) (Figure 2) made biodiversity contribute less to virus discovery in the three regions than in the world (Zhang et al., 2020). With exposure to a higher density of mammals played a slightly larger role in virus discovery in Africa than in China and the United States (Appendix 3—figure 9 to Appendix 3—figure 11).

Our discovery probability maps for 2010–2019 in three regions captured most historical hotspots, though several small new areas in central-eastern and southwestern United States, eastern and western China, as well as northern Africa would also make greater contribution to virus discovery (Figure 6). Our model has a good predictive ability, given 84% (37/44) new virus species in 2010–2019 were discovered in high-risk areas we have defined—85% percentiles of discovery probability within each region. Further, 35% (13/37) of those viruses discovered in high-risk areas since 2010 were discovered at the potential new hotspots where there had not been any virus discoveries in the past.

Our subgroup analyses distinguishing viruses firstly discovered in regions and those that had been discovered elsewhere in the world suggested in both the United States and Africa, discoveries of viruses firstly discovered in regions were more likely to be associated with climatic and biodiversity variables while discoveries of viruses had been discovered elsewhere in the world were more likely to be associated with socio-economic variables. This is plausible, again because after a novel virus was discovered elsewhere in the world, it is usually areas with a higher socio-economic level that first capture the virus in the local region.

This study had limitations. First, one common problem for data collected from literature review is the time lag between virus discovery and publication, in which case the virus data are likely to be matched to covariates in later decades. Second, we acknowledge that it is possible we have not identified the earliest report for some well-known viruses such as yellow fever virus, measles virus, especially in the post-vaccination era. Third, we were unable to identify robust and comprehensive data for all three regions on virus discovery effort (e.g. government transparency, laboratory infrastructure and technology), although we interpret GDP and university count as being an indirect measure of resources available for this activity. Previous studies have tried to use the bibliographic data to correct for the discovery effort (; ). However, this strategy worked less well for our data as the frequency of published paper from virus-related scientific journals has only a weak link to publications on novel human-infective RNA virus (Appendix 3—figure 1).

The study adds to our previous study (Zhang et al., 2020) in several ways. First, we firstly construct data sets of human-infective RNA virus discovery reflecting the viral richness in three broad regions of the world. Second, we reduced the heterogeneity of the predictors by focusing on regions, including those predictors reflecting the research effort. Research effort is less variable within restricted regions and therefore has less effect on virus detection. This implies our predicted hotspots stand closer to the virus geographic distribution in nature. Third, the predicted hotspots derived from regional analysis have a higher precision than at a global scale, for example, specific areas in the United States and China were identified as hotspots from regional analysis, rather than the whole eastern area from the global analysis. This helps target areas for future surveillance.

In conclusion, a heterogeneous pattern of virus discovery-driver relationships was identified across three regions and the globe. Within regions virus discovery is driven more by land use and socio-economic variables; climate and biodiversity variables are consistently less important predictors than at a global scale. We mapped with good accuracy that in 2010–2019 three regions where human-infective RNA viruses had previously been discovered would continue to be the discovery hotspots, but in addition, several new areas in each region would make great contribution to virus discovery. Results from the study could guide active surveillance for new human-infective viruses in high-risk areas.

## Figures and Tables

**Appendix 3—figure 1.**
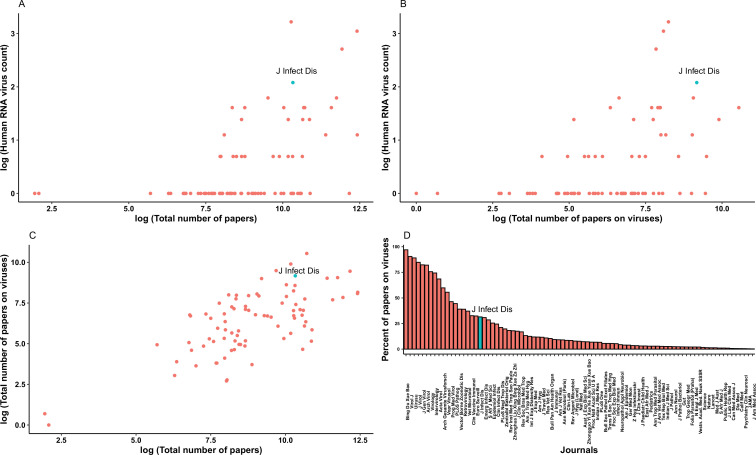
Relationship between published human-infective RNA virus count and total number of papers from the journals which published all human-infective RNA viruses in Web of Science. (A) Total number of papers vs. published human virus count; (B) Total number of papers on viruses vs. published human virus count; (C) Total number of papers vs. total number of papers on viruses; (D) Percent of papers on viruses in each journal. Journal of Infectious Diseases (JID) is highlighted in blue.

**Appendix 3—figure 2.**
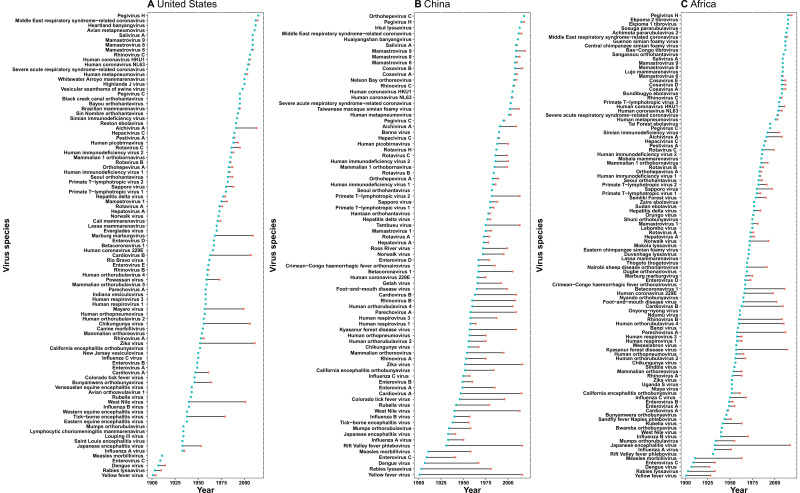
Time lag of human-infective RNA virus discovery between the three regions and the world. (A) United States. (B) China. (C) Africa. The blue dots represent the original discovery year of each virus in the world; the red dots represent the discovery year of each virus in three regions; and the segments between them represent the time lag.

**Appendix 3—figure 3.**
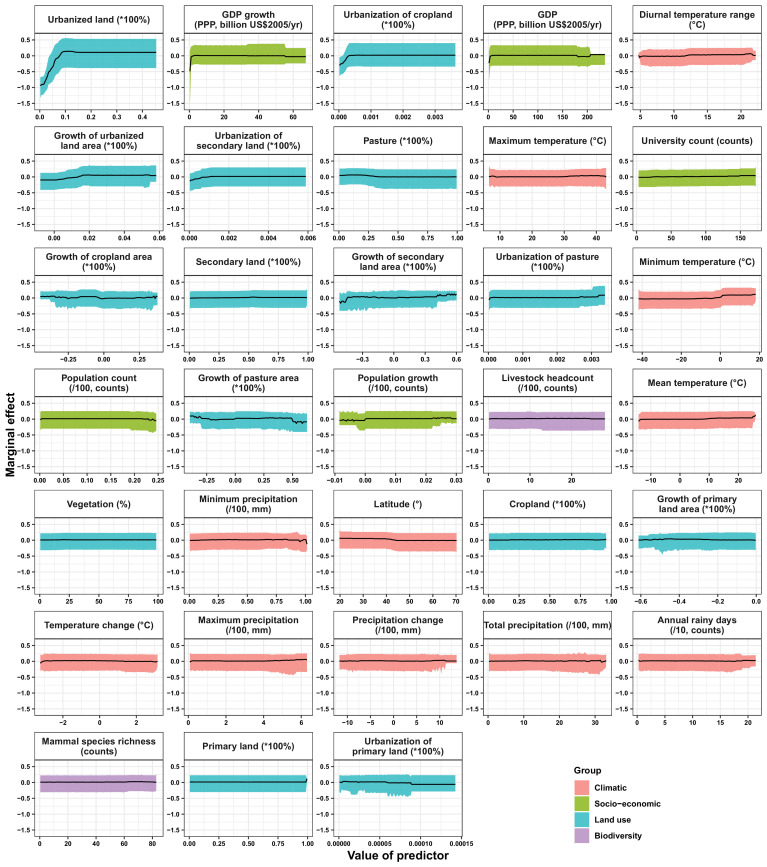
Partial dependence plots showing the influence on human-infective RNA virus discovery for all predictors in the Unites States. Partial dependence plots show the effect of an individual predictor over its range on the response after factoring out other predictors. Fitted lines represent the median (black) and 95% quantiles (coloured) based on 1000 replicated boosted regression tree models. Y axes are centred around the mean without scaling. X axes show the range of sampled values of predictors.

**Appendix 3—figure 4.**
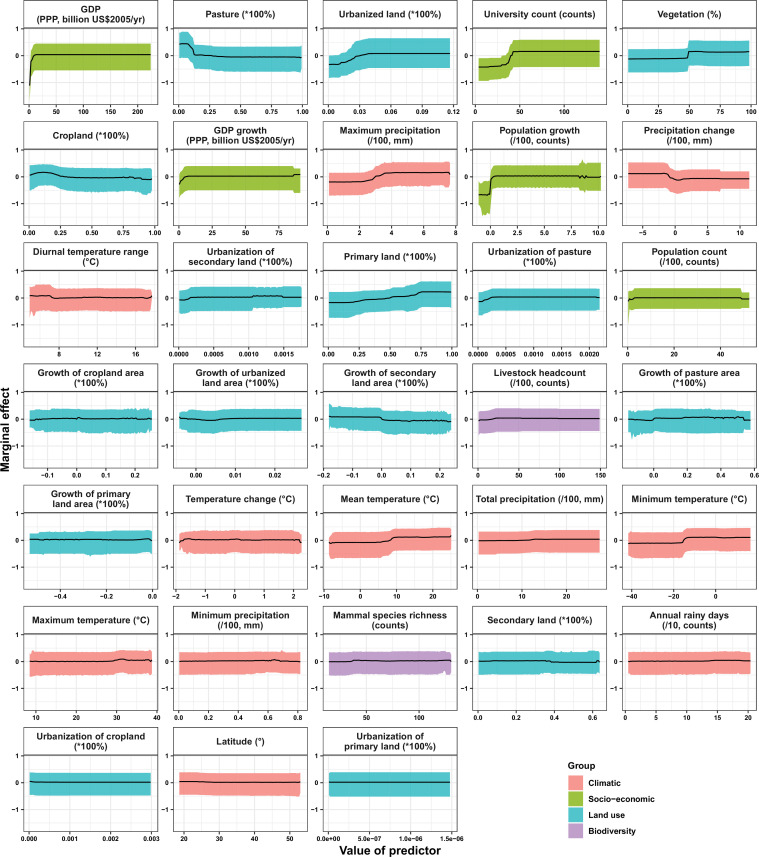
Partial dependence plots showing the influence on human-infective RNA virus discovery for predictors in China. Partial dependence plots show the effect of an individual predictor over its range on the response after factoring out other predictors. Fitted lines represent the median (black) and 95% quantiles (coloured) based on 1000 replicated boosted regression tree models. Y axes are centred around the mean without scaling. X axes show the range of sampled values of predictors.

**Appendix 3—figure 5.**
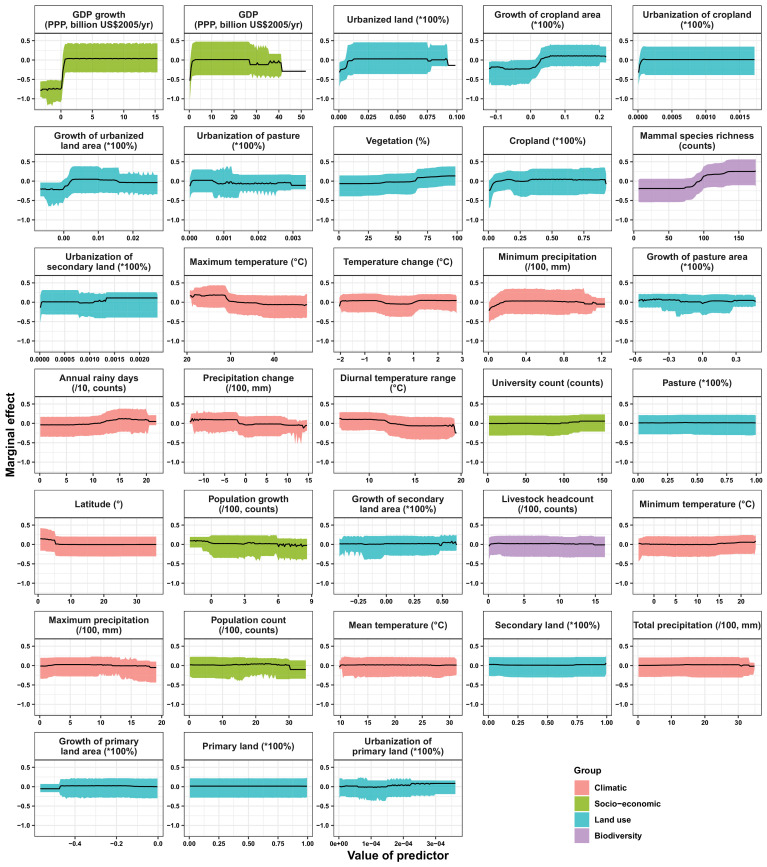
Partial dependence plots showing the influence on human-infective RNA virus discovery for all predictors in Africa. Partial dependence plots show the effect of an individual predictor over its range on the response after factoring out other predictors. Fitted lines represent the median (black) and 95% quantiles (coloured) based on 1000 replicated boosted regression tree models. Y axes are centred around the mean without scaling. X axes show the range of sampled values of predictors.

**Appendix 3—figure 6.**
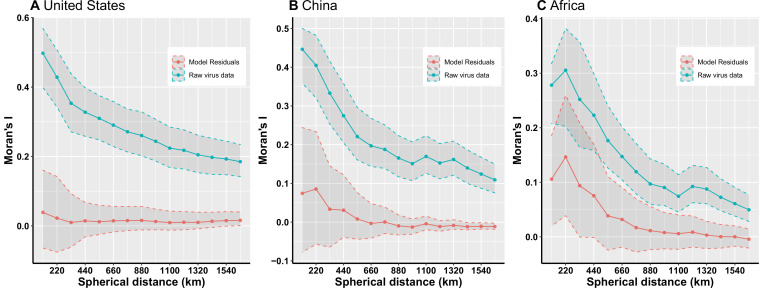
Moran’s I across different spherical distances. (A) United States; (B) China; (C) Africa. The solid line and dots represented the median Moran’s I value, and the grey area represented its 95% quantiles generated from 1000 samples (Blue: Raw virus data) or replicate boosted regression tree (BRT) models (Red: Model residuals). We used the fixed spherical distance as the neighbourhood weights—as there is no general consensus for selecting cut-off values, we chose spherical distances ranging from one time to fifteen times of distance of 1° grid cell at the equator, i.e. 110km to 1650km, considering the area of three regions. Our BRT models reduced Moran’s I value from a range of 0.19–0.50 for the raw virus data to 0.009–0.04 for the model residuals in the United States (A), 0.11–0.45 to –0.01–0.09 in China (B), 0.05–0.31 to –0.004–0.15 in Africa (C), suggesting that BRT models with 33 predictors have adequately accounted for spatial autocorrelations in the raw virus data in all three regions.

**Appendix 3—figure 7.**
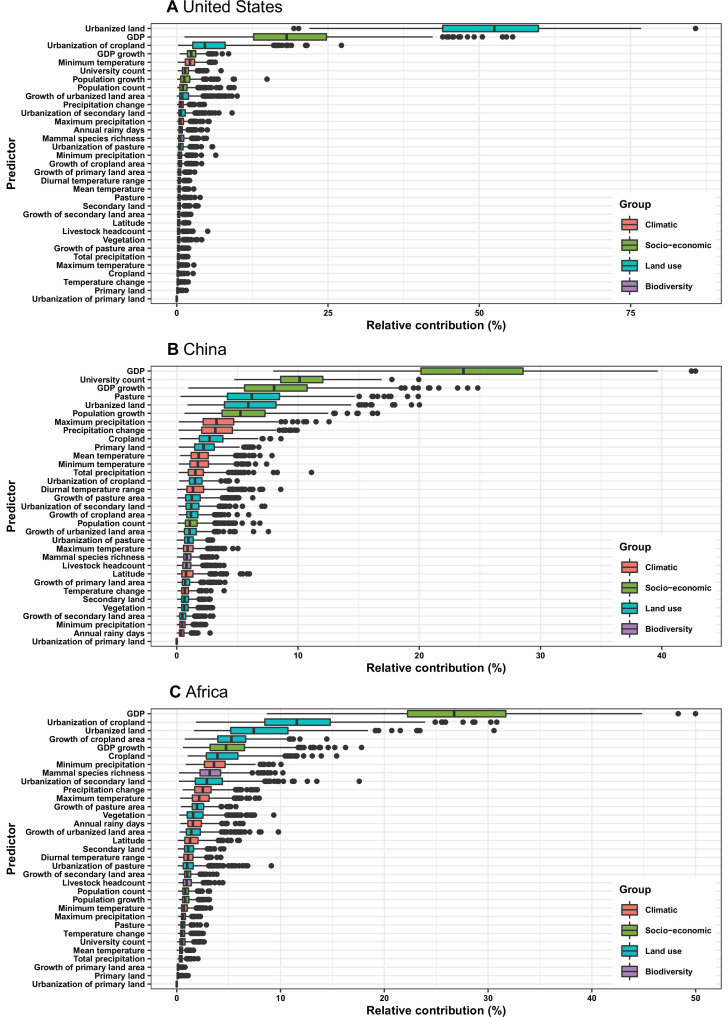
Relative contribution of predictors to human-infective RNA virus discovery in three regions. Virus discovery data were matched to time-varying covariate data by year. (A) United States. (B) China. (C) Africa. The boxplots show the median (black bar) and interquartile range (box) of the relative contribution across 1000 replicate boosted regression tree models, with whiskers indicating minimum and maximum and black dots indicating outliers.

**Appendix 3—figure 8.**
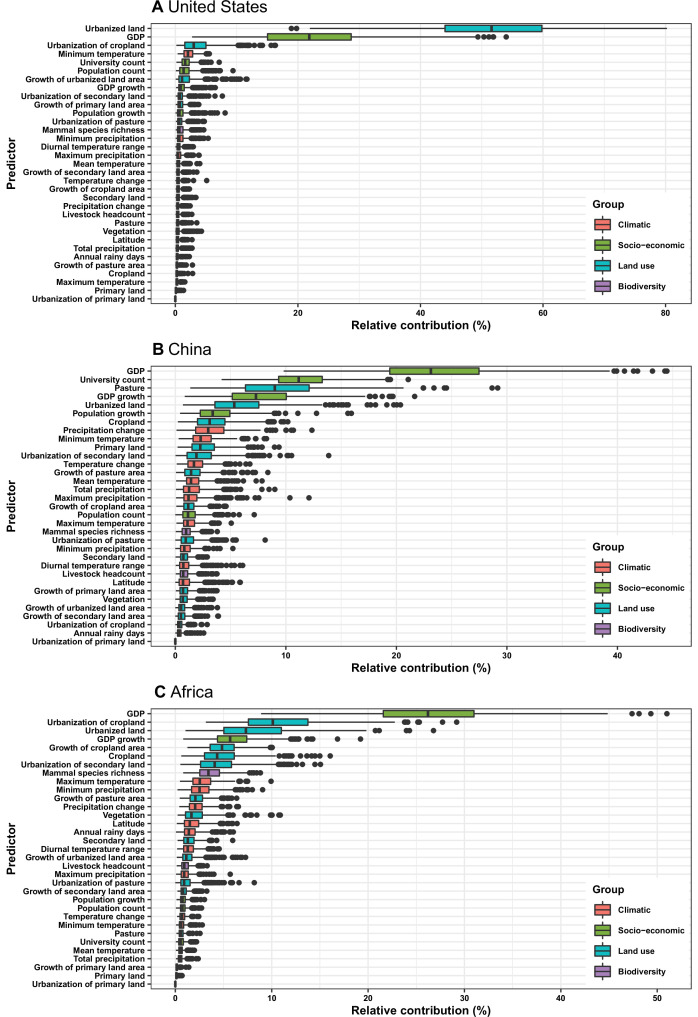
Relative contribution of predictors to human-infective RNA virus discovery in three regions. Virus discovery data at year t were matched to time-varying covariate data at year t-1. (A) United States. (B) China. (C) Africa. The boxplots show the median (black bar) and interquartile range (box) of the relative contribution across 1000 replicate boosted regression tree models, with whiskers indicating minimum and maximum and black dots indicating outliers.

**Appendix 3—figure 9.**
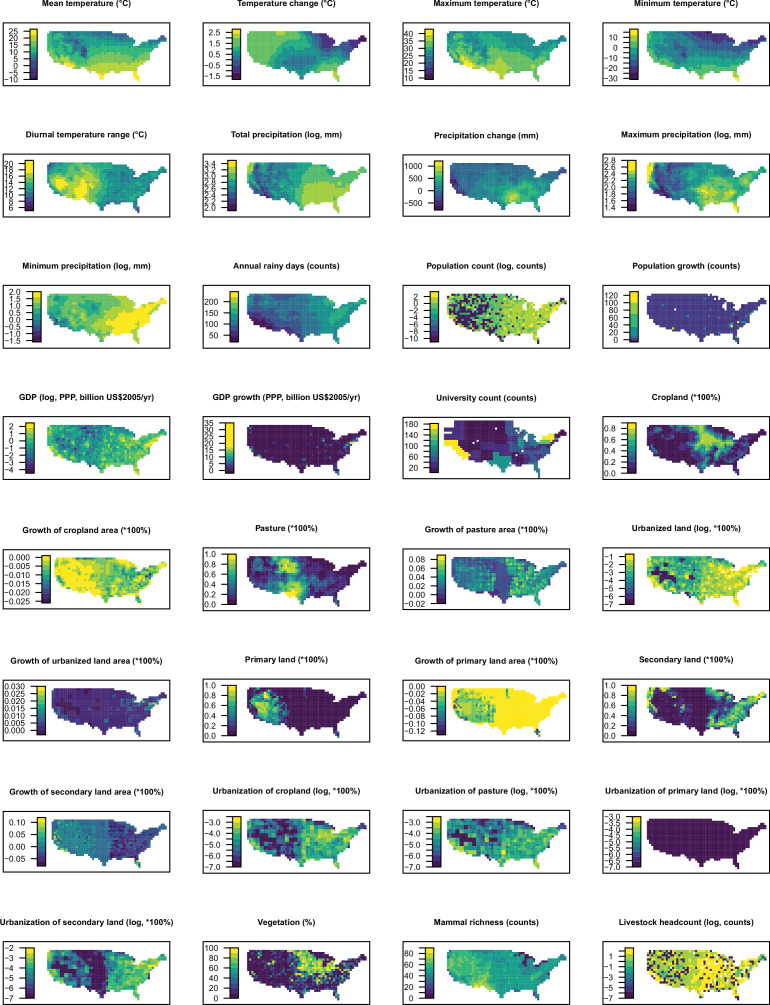
Distribution maps for 32 predictors in 2015 in the United States. The values of these explanatory variables and latitude in each grid cell were used to predict the virus discovery in the corresponding grid cell in the Unites States in 2010–2019. Explanatory variables were log transformed where necessary to get better visualization, not meaning they entered the model by logged values.

**Appendix 3—figure 10.**
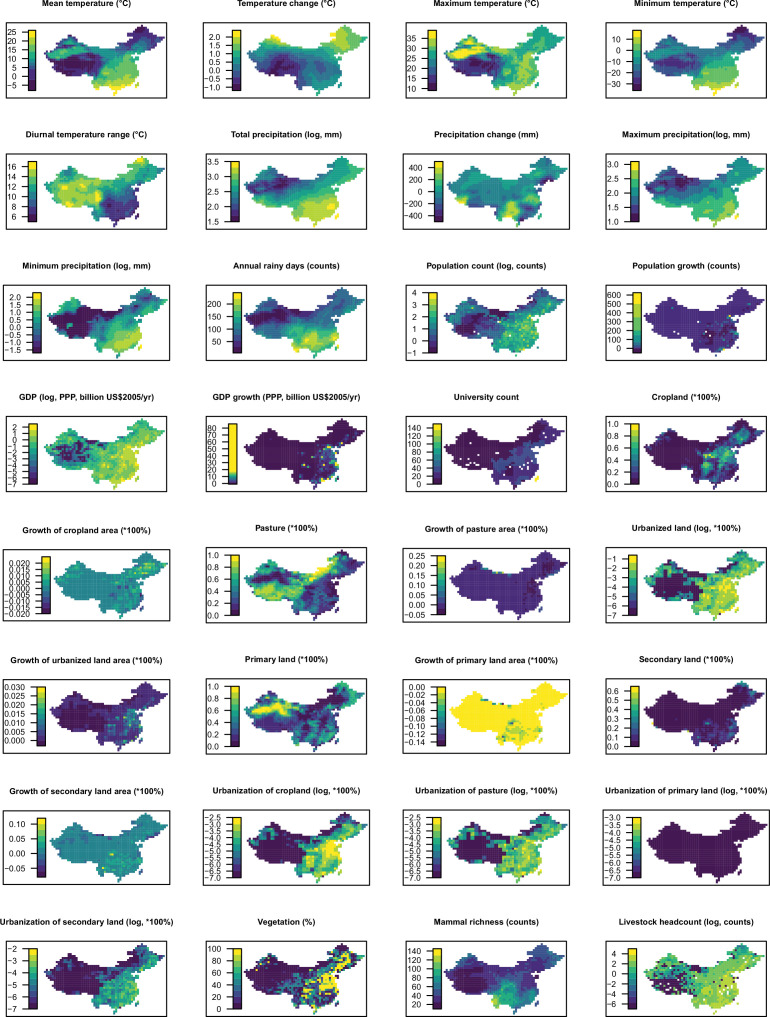
Distribution maps for 32 predictors in 2015 in China. The values of these explanatory variables and latitude in each grid cell were used to predict the virus discovery in the corresponding grid cell in China in 2010–2019. Explanatory variables were log transformed where necessary to get better visualization, not meaning they entered the model by logged values.

**Appendix 3—figure 11.**
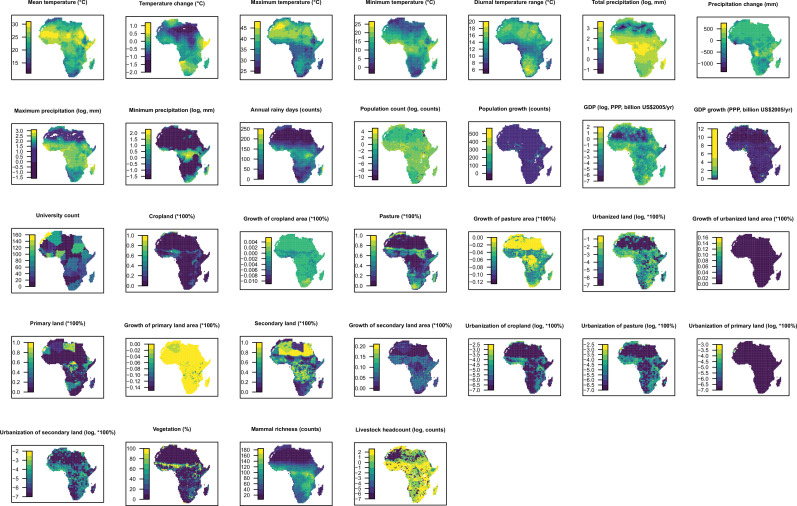
Distribution maps for 32 predictors in 2015 in Africa. The values of these explanatory variables and latitude in each grid cell were used to predict the virus discovery in the corresponding grid cell in Africa in 2010–2019. Explanatory variables were log transformed where necessary to get better visualization, not meaning they entered the model by logged values.

**Appendix 3—figure 12.**
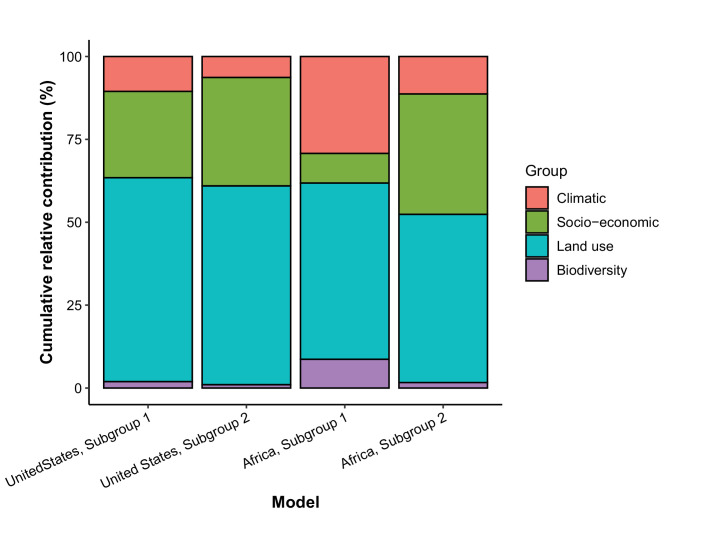
Cumulative relative contribution of predictors to human-infective RNA virus discovery by group in each model of subgroups. Subgroup 1 represents viruses firstly discovered from the region (United States or Africa); Subgroup 2 represents viruses firstly discovered elsewhere in the world. In the United States, virus count of Subgroup 1 and Subgroup 2 were 52 and 43, respectively. In Africa, virus count of Subgroup 1 and Subgroup 2 were 39 and 68, respectively. The relative contributions of all explanatory factors sum to 100% in each model, and each colour represents the cumulative relative contribution of all explan*atory factors within each group*.

## Data Availability

The authors confirm that all data or the data sources are provided in the paper and its Supplementary Materials. The final datasets and codes used for the analyses are available via figshare at https://doi.org/10.6084/m9.figshare.15101979. The following dataset was generated: ZhangF 2021Supporting data and R scripts for: Predictors of human RNA virus discovery in the United States, China and Africafigshare10.6084/m9.figshare.15101979PMC927895835666108 The following previously published dataset was used: WoolhouseMEJ BrierleyL 2017Epidemiological characteristics of human-infective RNA virusesEdinburgh DataShare10.7488/ds/2265PMC581947929461515

## Additional files

Transparent reporting form

## Appendix 1

**Appendix 1—table 1.** Summary of the human-infective RNA virus data sets in the United States, Africa, and China.

Species	Original discovery year	United States					China					Africa				
		Reported?	Discovery year	location	Lat	Lon	Reported?	Discovery year	location	Lat	Lon	Reported?	Discovery year	location	Lat	Lon
Argentinian mammarenavirus	1958	No					No					No				
Brazilian mammarenavirus	1994	Yes Barry et al., 1995	1995	New Haven, Connecticut	41.31	--72.93	No					No				
Cali mammarenavirus	1971	Yes Buchmeier et al., 1974	1974	Houston, Texas	29.76	--95.37	No					No				
Chapare mammarenavirus	2008	No					No					No				
Guanarito mammarenavirus	1991	No					No					No				
Lassa mammarenavirus	1970	Yes Buckley and Casals, 1970	1970	New Haven, Connecticut	41.31	--72.93	No					Yes Buckley and Casals, 1970	1970	Lassa, Borno State, Nigeria	10.69	13.27
Lujo mammarenavirus	2009	No					No					Yes Briese et al., 2009	2009	Lusaka, Zambia	--15.39	28.32
Lymphocytic choriomeningitis mammarenavirus	1934	Yes Armstrong and Lillie, 1934	1934	St. Louis county, Missouri	38.61	--90.41	No					No				
Machupo mammarenavirus	1964	No					No					No				
Mobala mammarenavirus	1985	No					No					Yes Georges et al., 1985	1985	Bouboui and Gomoka village, Boali town, Central African Republic	4.89	18.14
Whitewater Arroyo mammarenavirus	2000	Yes Enserink, 2000	2000	Alameda County, California	37.60	--121.72	No					No				
Mamastrovirus 1	1975	Yes Oshiro et al., 1981	1981	Martin County, California	40.22	--123.10	Yes Xu et al., 1981	1981	Guangzhou, Guangdong	23.13	113.26	Yes Dowling and Wynne, 1981	1981	Lebowa, South Africa	--23.5	29.5
Mamastrovirus 6	2008	Yes Finkbeiner et al., 2009c	2009	St. Louis, Missouri	38.63	--90.20	Yes Chu et al., 2010	2010	Hong Kong	22.40	114.11	Yes Kapoor et al., 2009	2009	Maiduguri, Borno State, Nigeria	11.83	13.15
Mamastrovirus 8	2009	Yes Finkbeiner et al., 2009a	2009	St. Louis, Missouri	38.63	--90.20	Yes Wang et al., 2013	2013	Nanjing, Jiangsu and Lanzhou, Gansu	31.95	118.78	Yes Kapoor et al., 2009	2009	Maiduguri, Borno State, Nigeria	11.83	13.15
Mamastrovirus 9	2009	Yes Finkbeiner et al., 2009b	2009	Accomack and Northampton Counties, Virginia	37.71	--75.81	Yes Tao et al., 2019	2019	Jinan, Shandong	36.68	117.11	Yes Kapoor et al., 2009	2009	Maiduguri, Borno State, Nigeria	11.83	13.15
Mammalian 1 orthobornavirus	1985	Yes Rott et al., 1985	1985	Philadelphia, Pennsylvania	39.95	--75.17	Yes Chen et al., 1999	1999	Taiwan	23.70	120.96	Yes Bode et al., 1992	1992	Rural area of East Africa	--1.28	34.53
Mammalian 2 orthobornavirus	2015	No					No					No				
Norwalk virus	1972	Yes Kapikian et al., 1972	1972	Norwalk, Ohio	41.24	--82.62	Yes Fang et al., 1995	1995	Henan	33.88	113.48	Yes Taylor et al., 1993	1993	Pretoria, Gauteng province, South Africa	--25.75	28.23
Sapporo virus	1980	Yes Nakata et al., 1988	1988	Houston, Texas	29.76	--95.37	Yes Nakata et al., 1988	1988	Shanghai	31.23	121.47	Yes Wolfaardt et al., 1997	1997	Pretoria, Gauteng province, South Africa	--25.75	28.23
Vesicular exanthema of swine virus	1998	Yes Smith et al., 1998	1998	Corvallis, Oregon	44.56	--123.26	No					No				
Alphacoronavirus 1	2007	No					No					No				
Human coronavirus 229E	1966	Yes Hamre and Procknow, 1966	1966	Chicago, Illinois	41.88	--87.63	Yes Virus Research Group of Kun Number 323 Unit, The Chinese People’s Liberation Army, 1975	1975	Kunming, Yunnan	25.07	102.68	Yes Hays and Myint, 1998	1998	Kumasi, Ghana	6.70	--1.62
Human coronavirus NL63	2004	Yes Esper et al., 2005	2005	New Haven, Connecticut	41.31	--72.93	Yes Chan et al., 2005	2005	Hong Kong	22.40	114.11	Yes Smuts et al., 2008	2008	Cape Town, Western Cape Province, South Africa	--33.90	18.57
Betacoronavirus 1	1967	Yes McIntosh et al., 1967	1967	Bethesda, Maryland	38.98	--77.09	Yes Chan et al., 2005	2005	Hong Kong	22.40	114.11	Yes Venter et al., 2011	2011	Pretoria, Gauteng province, South Africa	--25.75	28.23
Human coronavirus HKU1	2005	Yes Esper et al., 2006	2006	New Haven, Connecticut	41.31	--72.92	Yes Woo et al., 2005	2005	Hong Kong	22.40	114.11	Yes Venter et al., 2011	2011	Pretoria, Gauteng province, South Africa	--25.75	28.23
Middle East respiratory syndrome-related coronavirus	2012	Yes* Bialek et al., 2014	2014	Lake county, Indiana	41.45	--87.37	Yes* Gao and Song, 2015	2015	Huizhou, Guangdong	23.09	114.40	Yes* Abroug et al., 2014	2014	Monastir, Tunisia	35.79	10.82
Severe acute respiratory syndrome-related coronavirus	2003	Yes* Charles M, 2003	2003	Atlanta, Georgia	33.75	--84.39	Yes Peiris et al., 2003a	2003	Hong Kong	22.40	114.11	Yes Chiu et al., 2004	2004	Pretoria, Gauteng province, South Africa	--25.75	28.23
Human torovirus (been abolished)	1984	No					No					No				
Bundibugyo ebolavirus	2008	No					No					Yes Smuts et al., 2008	2008	Bundibugyo and Kikyo town, Bundibugyo District, Western Uganda	0.71	30.06
Reston ebolavirus	1991	Yes Miranda et al., 1991	1991	Reston, Fairfax County, Virginia	38.96	--77.35	No					No				
Sudan ebolavirus	1977	No					No					Yes Bowen et al., 1977	1977	Maridi, South Sudan	4.91	29.45
Tai Forest ebolavirus	1995	No					No					Yes Le Guenno et al., 1995	1995	Abidjan, Cote-d’lvoire	5.36	--4.01
Zaire ebolavirus	1977	No					No					Yes Johnson et al., 1977	1977	Yambuku village, Democratic Republic of the Congo	2.83	22.22
Marburg marburgvirus	1968	Yes* Centers for Disease Control and Prevention, 2009	2009	Denver county, Colorado	39.55	--105.78	No					Yes Gear et al., 1975	1975	Johannesburg, South Africa	--26.20	27.90
Aroa virus	1971	No					No					No				
Bagaza virus	2009	No					No					No				
Banzi virus	1959	No					No					Yes Smithburn et al., 1959	1959	Maponde's Kraal(Usutu river), South Africa	--26.52	31.67
Cacipacore virus	2011	No					No					No				
Dengue virus	1907	Yes Lavinder and Francis, 1914	1914	Savannah, Georgia	32.02	--81.12	Yes Clarke et al., 1967	1967	Southwest Taiwan	23.06	120.59	Yes Edington, 1927	1927	Durban, KwaZulu-Natal Province, South Africa	--29.86	31.02
Edge Hill virus	1985	No					No					No				
Gadgets Gully virus	1991	No					No					No				
Ilheus virus	1947	No					No					No				
Japanese encephalitis virus	1933	Yes* Perex-Pina and Merikangas, 1953	1953	Waltham, Massachusetts	42.38	--71.24	Yes Yen, 1941	1941	Beijing	40.01	116.41	Yes Simon-Loriere et al., 2017	2017	Cunene, Angola	--16.28	15.28
Kokobera virus	1964	No					No					No				
Kyasanur forest disease virus	1957	No					Yes Wang et al., 2009	2009	Hengduanshan Mountain, Yunnan	27.50	99.00	Yes Andayi et al., 2014	2014	Djibouti, Republic of Djibouti	11.57	43.15
Langat virus	1956	No					No					No				
Louping ill virus	1934	Yes Rivers and Schwentker, 1934	1934	New York	40.71	--74.01	No					No				
Murray Valley encephalitis virus	1952	No					No					No				
Ntaya virus	1952	No					No					Yes Smithburn, 1952	1952	Bwamba county, Uganda	0.75	30.02
Omsk hemorrhagic fever virus	1948	No					No					No				
Powassan virus	1959	Yes Goldfield et al., 1973	1973	Middlesex County, New Jersey	40.54	--74.37	No					No				
Rio Bravo virus	1962	Yes Suklin et al., 1962	1962	Dallas city, Texas	32.78	--96.80	No					No				
Saint Louis encephalitis virus	1933	Yes Webster and Fite, 2009	1933	St. Louis City, Missouri	38.63	--90.20	No					No				
Tembusu virus	1975	No					Yes Tang et al., 2013	2013	Shandong	36.40	118.77	No				
Tick-borne encephalitis virus	1938	Yes* Cruse et al., 1979	1979	Cleveland, Ohio	41.51	--81.69	Yes Wang and Zhao, 1956	1956	Bali village, Wuchang, Heilongjiang	44.91	127.16	No				
Uganda S virus	1952	No					No					Yes Dick and Haddow, 1952	1952	Bwamba county, Uganda	0.75	30.02
Usutu virus	2009	No					No					No				
Wesselsbron virus	1957	No					No					Yes Smithburn et al., 1957	1957	Lake Simbu region, Maputaland, KwaZulu-Natal, South Africa	--27.36	32.32
West Nile virus	1940	Yes Nash et al., 2001	2001	New York	40.71	--74.01	Yes Li et al., 2013	2013	Jiashi County, Xinjiang	39.58	77.18	Yes Smithburn et al., 1940	1940	Omogo, West Nile district, Uganda	0.42	33.21
Yellow fever virus	1901	Yes Guiteras, 1904	1904	Laredo, Texas	27.51	--99.51	Yes* Chen and Lu, 2016	2016	Beijing	40.01	116.41	Yes Stokes et al., 1928	1928	Larteh, Ghana	5.94	--0.07
Zika virus	1952	Yes* Foy et al., 2011	2011	Northern Colorado	39.55	--105.78	Yes* Sun et al., 2016	2016	Gan County, Ganzhou city, Jiangxi	25.86	115.02	Yes Dick, 1952	1952	Zika, Uganda	0.12	32.53
Hepacivirus C	1989	Yes Choo et al., 1989	1989	Emeryville, California	37.83	--122.29	Yes Xu et al., 1990a	1990	Qidong county, Jiangsu	31.88	121.72	Yes Kew et al., 1990	1990	Johannesburg, South Africa	--26.20	27.90
Pegivirus C	1995	Yes Simons et al., 1995	1995	Chapel Hill, North Carolina; Rochester, Minnesota; Dallas, Texas	35.91	--79.06	Yes Wang et al., 1996	1996	Beijing	40.01	116.41	Yes Simons et al., 1995	1995	Cairo, Egypt	30.04	31.24
Pegivirus H	2015	Yes Kapoor et al., 2015	2015	New York city, New York	40.71	--74.01	Yes Wang et al., 2018	2018	Guangzhou, Guangdong	23.13	113.26	Yes Rodgers et al., 2019	2019	Ebolowa, Cameroon	2.92	11.15
Pestivirus A	1988	Yes Yolken et al., 1989	1989	Whiteriver, Arizona	33.83	--109.97	No					Yes Giangaspero et al., 1988	1988	Zambia	--13.13	27.85
Andes orthohantavirus	1996	No					No					No				
Bayou orthohantavirus	1995	Yes Morzunov et al., 1995	1995	Louisiana	30.98	--91.96	No					No				
Black creek canal orthohantavirus	1995	Yes Ravkov et al., 1995	1995	Miami-Dade County, Florida	25.76	--80.34	No					No				
Choclo orthohantavirus	2000	No					No					No				
Dobrava-Belgrade orthohantavirus	1992	No					No					No				
Hantaan orthohantavirus	1978	No					Yes Lee et al., 1980	1980	Zhejiang	29.14	119.79	No				
Laguna Negra orthohantavirus	1997	No					No					No				
Puumala orthohantavirus	1980	No					No					No				
Sangassou orthohantavirus	2010	No					No					Yes Klempa et al., 2010	2010	Sangassou village, Macenta district, Forest Guinea	8.24	--9.32
Seoul orthohantavirus	1982	Yes Forthal et al., 1987	1987	Mississippi	32.57	--89.88	Yes Song et al., 1982	1982	Jiangsu	33.14	119.79	Yes Tomori et al., 1986	1986	Jos, Nigeria	9.90	8.86
Sin Nombre orthohantavirus	1993	Yes Nichol et al., 1993	1993	New Mexico	34.52	--105.87	No					No				
Thailand orthohantavirus	2006	No					No					No				
Thottopalayam thottimvirus	2007	No					No					No				
Tula orthohantavirus	1996	No					No					No				
Orthohepevirus A	1983	Yes* De Cock et al., 1987	1987	Los Angeles County, California	34.05	--118.24	Yes Huang et al., 1989	1989	Kashi county, Kashi city, Xinjiang	39.46	75.99	Yes Belabbes et al., 1985	1985	Medea town, Algeria	36.26	2.75
Orthohepevirus C	2018	No					Yes Sridhar et al., 2018	2018	Hong Kong	22.40	114.11	No				
Crimean-Congo haemorrhagic fever orthonairovirus	1967	No					Yes Yen et al., 1985	1985	Bachu, southern Xinjiang	39.79	78.55	Yes Simpson et al., 1967	1967	Kisangani, Tshopo province, Democratic Republic of the Congo	0.53	25.19
Dugbe orthonairovirus	1969	No					No					Yes Causey et al., 1969	1969	Ibadan, Nigeria	7.35	3.88
Nairobi sheep disease orthonairovirus	1969	No					No					Yes Morrill et al., 1991	1991	Mombasa; Malindi; and Kilifi, Coast Province, Kenya	--3.34	39.57
Thiafora orthonairovirus	1989	No					No					No				
Influenza A virus	1933	Yes Francis and Magill, 1935	1935	Philadelphi, Pennsylvania	39.95	--75.17	Yes Chang and Chiang, 1950	1950	Beijing	40.01	116.41	Yes Isaacs and Andrews, 1951	1951	Johannesburg, South Africa and Cape Town, South Africa	--26.20	27.90
Influenza B virus	1940	Yes Francis, 1940	1940	Irvington village, Greenburgh town, Westchester County, New York	41.03	--73.87	Yes Wen and Chu, 1957	1957	Beijing	40.01	116.41	Yes Montefiore et al., 1970	1970	Arusha, Arusha Region, Tanzania	--3.37	36.69
Influenza C virus	1950	Yes Francis et al., 1950	1950	Ann Arbor city, Michigan	42.28	--83.74	Yes Zhang, 1957	1957	Beijing	40.01	116.41	Yes Joosting et al., 1968	1968	Johannesburg, South Africa	--26.20	27.90
Dhori thogotovirus	1985	No					No					No				
Thogoto thogotovirus	1969	No					No					Yes Causey et al., 1969	1969	Ibadan, Nigeria	7.35	3.88
Avian orthoavulavirus 1	1943	Yes Burnet, 1943	1943	Washington, D. C.	38.91	--77.04	No					No				
Hendra henipavirus	1995	No					No					No				
Nipah henipavirus	1999	No					No					No				
Canine morbillivirus	1955	Yes Karzon, 1955	1955	Buffalo, New York	42.89	--78.88	No					No				
Measles morbillivirus	1911	Yes Goldberger and Anderson, 1911	1911	Washington, D. C.	38.91	--77.04	Yes Tang et al., 1958	1958	Beijing	40.01	116.41	Yes Baylet et al., 1963	1963	Dakar, Senegal	14.72	--17.47
Human respirovirus 1	1958	Yes Chanock et al., 1958	1958	Washington, D. C.	38.91	--77.04	Yes Chen et al., 1964	1964	Zhejiang	29.14	119.79	Yes Taylor-Robinson and Tyrrell, 1963	1963	Cape Town, Western Cape Province, South Africa	--33.90	18.57
Human respirovirus 3	1958	Yes Chanock et al., 1958	1958	Washington, D. C.	38.91	--77.04	Yes Yu et al., 1987	1987	Guangzhou, Guangdong	23.13	113.26	Yes Taylor-Robinson and Tyrrell, 1963	1963	Cape Town, Western Cape Province, South Africa	--33.90	18.57
Achimota pararubulavirus 2	2013	No					No					Yes Baker et al., 2013	2013	Volta, Ghana	6.05	0.37
Human orthorubulavirus 2	1956	Yes Chanock, 1956	1956	Cincinnati, Ohio	39.10	--84.51	Yes Pathogen biology research group, Jiangsu new medical college, 1975	1975	Nanjing, Jiangsu	31.95	118.78	Yes Balestrieri et al., 1967	1967	Accra, Ghana	5.60	--0.19
Human orthorubulavirus 4	1960	Yes Johnson et al., 1960	1960	Bethesda, Maryland	38.98	--77.09	Yes Lau et al., 2005	2005	Hong Kong	22.40	114.11	Yes Niang et al., 2010	2010	Ndiop village, Sine Saloum region, Senegal	15.18	--16.74
Mammalian orthorubulavirus 5	1959	Yes Schultz and Habel, 1959	1959	Stanford, California	37.42	--122.17	No					No				
Menangle pararubulavirus	1998	No					No					No				
Mumps orthorubulavirus	1934	Yes Johnson and Goodpasture, 1934	1934	Nashville, Tennessee	36.16	--86.78	Yes Wang et al., 1958	1958	Beijing	40.01	116.41	Yes Bayer and Gear, 1955	1955	Johannesburg, South Africa	--26.20	27.90
Simian orthorubulavirus	1968	No					No					No				
Sosuga pararubulavirus	2014	No					No					Yes Albariño et al., 2014	2014	ˉ	3.76	32.82
Tioman pararubulavirus	2007	No					No					No				
Bunyamwera orthobunyavirus	1946	Yes Work, 1964	1964	Southern Florida	26.92	--81.21	No					Yes Smithburn et al., 1946	1946	Bwamba County, Uganda	0.75	30.02
Bwamba orthobunyavirus	1941	No					No					Yes Smithburn et al., 1941	1941	Bwamba county, Western Province of Uganda	0.75	30.02
California encephalitis orthobunyavirus	1952	Yes Hammon and Reeves, 1952	1952	Kern county, California	35.49	--118.86	Yes Gu et al., 1984	1984	Longhua, Shanghai	31.22	121.43	Yes Bardos and Sefcovicova, 1961	1961	Uganda	1.37	32.29
Caraparu orthobunyavirus	1961	No					No					No				
Catu orthobunyavirus	1961	No					No					No				
Guama orthobunyavirus	1961	No					No					No				
Guaroa orthobunyavirus	1959	No					No					No				
Kairi orthobunyavirus	1967	No					No					No				
Madrid orthobunyavirus	1964	No					No					No				
Marituba orthobunyavirus	1961	No					No					No				
Nyando orthobunyavirus	1965	No					No					Yes Williams et al., 1965	1965	Kisumu, Kenya	--0.09	34.77
Oriboca orthobunyavirus	1961	No					No					No				
Oropouche orthobunyavirus	1961	No					No					No				
Patois orthobunyavirus	1972	No					No					No				
Shuni orthobunyavirus	1975	No					No					Yes Moore et al., 1975	1975	Ibadan, Nigeria	7.38	3.95
Tacaiuma orthobunyavirus	1967	No					No					No				
Wyeomyia orthobunyavirus	1965	No					No					No				
Candiru phlebovirus	1983	No					No					No				
Punta Toro phlebovirus	1970	No					No					No				
Rift Valley fever phlebovirus	1931	No					Yes* Liu et al., 2016	2016	Beijing	40.01	116.41	Yes Daubney et al., 1931	1931	Rift Valley of Kenya Colony	--0.28	36.07
Sandfly fever Naples phlebovirus	1944	No					No					Yes Sabin, 1951	1951	Cairo, Egypt	30.04	31.24
Heartland banyangvirus	2012	Yes McMullan et al., 2012	2012	Andrew and Nodaway Counties, Missouri	39.82	--94.59	No					No				
Huaiyangshan banyangvirus	2011	No					Yes Zhang et al., 2011	2011	Huaiyangshan	31.37	115.39	No				
Uukuniemi phlebovirus	1970	No					No					No				
Human picobirnavirus	1988	Yes Grohmann et al., 1993	1993	Atlanta, Georgia	33.75	--84.39	Yes Rosen et al., 2000	2000	Lulong County, Hebei	39.94	116.94	No				
Equine rhinitis A virus	1962	No					No					No				
Foot-and-mouth disease virus	1965	No					Yes Luo et al., 1999	1999	Guangzhou	23.13	113.26	Yes Donia and Youssef, 2002	2002	Alexandria Governorate, Egypt	30.74	29.74
Cardiovirus A	1947	Yes Jonkers, 1961	1961	New Orleans, Louisiana	29.95	--90.07	Yes Feng et al., 2015	2015	Changchun, Jilin	43.87	125.34	Yes Dick and Best, 1948	1948	Entebbe, Uganda	0.05	32.46
Cardiovirus B	1963	Yes Jones et al., 2007	2007	San Diego, California	32.72	--117.16	Yes Cheng et al., 2009a	2009	Lanzhou, Gansu	36.06	103.79	Yes Zoll et al., 2009	2009	Cameroon	5.03	12.40
Cosavirus A	2008	No					Yes Dai et al., 2010	2010	Shanghai	31.23	121.47	Yes Kapusinszky et al., 2012	2012	Maiduguri, Borno State, Nigeria	11.83	13.15
Cosavirus B	2008	No					Yes Yang et al., 2016	2016	Zhenjiang, Jiangsu	32.19	119.43	No				
Cosavirus D	2008	No					No					Yes Kapusinszky et al., 2012	2012	Maiduguri, Borno State, Nigeria	11.83	13.15
Cosavirus E	2008	No					No					Yes Kapusinszky et al., 2012	2012	Maiduguri, Borno State, Nigeria	11.83	13.15
Cosavirus F	2012	No					No					No				
Enterovirus A	1949	Yes Sickles and Dalldorf, 1949	1949	New York	43.30	--74.22	Yes Xiao et al., 1985	1985	Tianjin	39.34	117.36	Yes Bayer and Gear, 1955	1955	Johannesburg, South Africa	--26.20	27.90
Enterovirus B	1949	Yes Sickles and Dalldorf, 1949	1949	Wilmington	39.74	--75.54	Yes Wu et al., 1960	1960	Fuzhou, Fujian	26.07	119.30	Yes Patz et al., 1953	1953	Middelburg, Transvaal, South Africa	--25.77	29.46
Enterovirus C	1909	Yes Flexner and Lewis, 1909	1909	New York city, New York	40.71	--74.01	Yes Yen and Hsü, 1941	1941	Bejing	39.90	116.41	Yes Hudson and Lennette, 1933	1933	Monrovia, Liberia	6.29	--10.76
Enterovirus D	1967	Yes Schieble et al., 1967	1967	Berkeley, California	37.87	--122.27	Yes Shanghai Eye and Skin Disease Prevention and Treatment Institute, 1979	1979	Shanghai	31.23	121.47	Yes Mirkovic et al., 1973	1973	Morocco	31.79	--7.09
Enterovirus E	1961	Yes Moscovivci et al., 1961	1961	Denver, Colorado	39.74	--104.99	No					No				
Enterovirus H	1965	No					No					No				
Rhinovirus A	1953	Yes Price, 1956	1956	Baltimore, Maryland	39.29	--76.61	Yes Guangzhou Institute of Medicine and Health, 1975	1975	Guangzhou, Guangdong	23.13	113.26	Yes Taylor-Robinson, 1963	1963	Cape Town, Western Cape Province, South Africa	--33.90	18.57
Rhinovirus B	1960	Yes Hamre and Procknow, 1961	1961	Chicago, Illinois	41.88	--87.63	Yes Xiang et al., 2008	2008	Beijing	40.01	116.41	Yes Briese et al., 2008	2008	Pretoria, Gauteng province, South Africa	--25.75	28.23
Rhinovirus C	2006	Yes Lamson et al., 2006	2006	New York city, New York	40.71	--74.01	Yes Lau et al., 2007	2007	Hong Kong	22.40	114.11	Yes Briese et al., 2008	2008	Pretoria, Gauteng province, South Africa	--25.75	28.23
Erbovirus A	2005	No					No					No				
Hepatovirus A	1973	Yes Feinstone et al., 1973	1973	Bethesda, Maryland	38.98	--77.09	Yes Microbiology Research Group of Shanghai First Medical College and Laboratory of Shanghai Sixth People’s Hospital, 1978	1978	Shanghai	31.23	121.47	Yes Szmuness et al., 1977	1977	Dakar, Senegal	14.72	--17.47
Aichivirus A	1991	Yes Chhabra et al., 2013	2013	Cincinnati, Ohio	39.10	--84.51	Yes Yang et al., 2009	2009	Shanghai	31.23	121.47	Yes Sdiri-Loulizi et al., 2008	2008	Monastir, Tunisia	35.77	10.82
Parechovirus A	1958	Yes Ramoz-alverz and Sabin, 1958	1958	Cincinnati, Ohio	39.10	--84.51	Yes Shan et al., 2009	2009	Shanghai	31.23	121.47	Yes Kapusinszky et al., 2012	2012	Ouagadougou, Burkina Faso	12.24	--1.56
Parechovirus B	2003	No					No					No				
Salivirus A	2009	Yes Greninger et al., 2009	2009	Northern California	38.84	--120.90	Yes Shan et al., 2010	2010	Shanghai	31.23	121.47	Yes Li et al., 2009	2009	Maiduguri, Borno State, Nigeria	11.83	13.15
Avian metapneumovirus	2011	Yes Kayali et al., 2011	2011	Memphis, Tennessee	35.15	--90.05	No					No				
Human metapneumovirus	2001	Yes Falsey et al., 2003	2003	Rochester, New York	43.16	--77.61	Yes Peiris et al., 2003b	2003	Hong Kong	22.40	114.11	Yes Madhi et al., 2003	2003	Johannesburg, South Africa	--26.20	27.90
Human orthopneumovirus	1957	Yes Chanock et al., 1957	1957	Baltimore, Maryland	39.29	--76.61	Yes Kun Number 323 Unit, the Chinese People’s Liberation Army, 1975	1975	Kunming, Yunnan	25.07	102.68	Yes Doggett, 1965	1965	Cape Town, Western Cape Province, South Africa	--33.90	18.57
Colorado tick fever virus	1946	Yes Florio et al., 1946	1946	Denver, Colorado	39.74	--104.99	Yes Yang et al., 1996	1996	Nanjing, Jiangsu	31.95	118.78	No				
Eyach virus	1980	No					No					No				
Corriparta virus	1967	No					No					No				
Great Island virus	1963	No					No					No				
Lebombo virus	1975	No					No					Yes Moore et al., 1975	1975	lbadan, Nigeria	7.38	3.95
Orungo virus	1976	No					No					Yes Tomori et al., 1976	1976	Ibadan, Nigeria	7.38	3.95
Mammalian orthoreovirus	1954	Yes Ramos-Alvarez and Sabin, 1954	1954	Cincinnati, Ohio	39.10	--84.51	Yes Zhao et al., 1995	1995	Xuzhou, Jiangsu	34.26	117.19	Yes Malherbe et al., 1963	1963	Johannesburg, South Africa	--26.20	27.90
Nelson Bay orthoreovirus	2007	No					Yes* Cheng et al., 2009b	2009	Hong Kong	22.40	114.11	No				
Rotavirus A	1973	Yes Kapikian et al., 1976	1976	Washington, D. C.	38.90	--77.04	Yes PaPa et al., 1979	1979	Beijing	40.01	116.41	Yes Tomori et al., 1976	1976	Johannesburg, South Africa	--26.20	27.90
Rotavirus B	1984	Yes Eiden et al., 1985	1985	Baltimore, Maryland	39.29	--76.61	Yes Hung et al., 1984	1984	Jinzhou, Liaoning	41.10	121.13	Yes Nakata et al., 1987	1987	Kenya	--0.02	37.91
Rotavirus C	1986	Yes Jiang et al., 1995	1995	Providence, Rhode Island	41.82	--71.41	Yes Qiao et al., 1999	1999	Beijing	40.01	116.41	Yes Sebata and Steele, 1999	1999	Pretoria, Gauteng province, South Africa	--25.75	28.23
Rotavirus H	1987	No					Yes Wang et al., 1987	1987	Huaihua, Hunan Province	27.55	109.96	No				
Banna virus	1990	No					Yes Xu et al., 1990b	1990	Xishuangbanna, Yunnan Province	21.90	100.80	No				
Primate T-lymphotropic virus 1	1980	Yes Poiesz et al., 1980	1980	Bethesda, Maryland	38.98	--77.09	Yes Hung et al., 1984	1984	Shenyang, Liaoing	41.80	123.38	Yes Williams et al., 1984	1984	Ibadan, Nigeria	7.38	3.95
Primate T-lymphotropic virus 2	1982	Yes Kalyanaraman et al., 1982	1982	Seattle, Washington	47.61	--122.33	Yes Ma et al., 2013	2013	Henan and Hubei	32.21	112.96	Yes Delaporte et al., 1991	1991	Franceville, Gabon	--1.63	13.60
Primate T-lymphotropic virus 3	2005	No					No					Yes Calattini et al., 2005	2005	Océan department, South Province, Cameroon	2.50	10.50
Human immunodeficiency virus 1	1983	Yes Safai et al., 1984	1984	Washington, D. C.	38.90	--77.04	Yes Chang et al., 1986	1986	Hong Kong	22.40	114.11	Yes Brun-Vézinet et al., 1984	1984	Kisangani, Tshopo province, Democratic Republic of the Congo	0.53	25.19
Human immunodeficiency virus 2	1986	Yes* Centers for Disease Control, 1988	1988	New Jersey	40.06	--74.41	Yes* Yan et al., 2000	2000	Fuzhou, Fujian	26.07	119.30	Yes Kanki et al., 1986	1986	Dakar, Senegal	14.72	--17.47
Simian immunodeficiency virus	1992	Yes Khabbaz et al., 1992	1992	Atlanta, Georgia	33.75	--84.39	No					Yes Calattini et al., 2005	2005	Cameroon	7.37	12.35
Central chimpanzee simian foamy virus	2012	No					No					Yes Rua et al., 2012	2012	Near Dja Nature Reserves, Southern Cameroon	4.50	13.50
Eastern chimpanzee simian foamy virus	1971	No					No					Yes Achong et al., 1971	1971	Kenya	--0.02	37.91
Grivet simian foamy virus	1997	No					No					No				
Guenon simian foamy virus	2012	No					No					Yes Rua et al., 2012	2012	Near lolodrof, Southern Cameroon	3.23	10.73
Taiwanese macaque simian foamy virus	2002	No					Yes Huang et al., 2012	2012	Yunnan	25.18	101.86	No				
Australian bat lyssavirus	1998	No					No					No				
Duvenhage lyssavirus	1971	No					No					Yes Meredith et al., 1971	1971	Pretoria, Gauteng province, South Africa	--25.75	28.23
European bat Yeslyssavirus	1989	No					No					No				
European bat 2 lyssavirus	1986	No					No					No				
Irkut lyssavirus	2013	No					Yes Liu et al., 2013	2013	Tonghua county, Jilin	41.68	125.76	No				
Mokola lyssavirus	1972	No					No					Yes Familusi et al., 1972	1972	Ibadan, Nigeria	7.38	3.95
Rabies lyssavirus	1903	Yes Black and Powers, 1910	1910	Southern California	34.57	--116.76	Yes Wu, 1981	1981	Beijing	40.01	116.41	Yes Wilhelm and Alexis, 1933	1933	Carolina, Mpumalanga, South Africa	--26.07	30.12
Bas-Congo tibrovirus	2012	No					No					Yes Grard et al., 2012	2012	Mangala village, Boma Bungu Health Zone, Democratic Republic of Congo (DRC)	--4.04	21.76
Ekpoma Yestibrovirus	2015	No					No					Yes Stremlau et al., 2015	2015	Irrua, Edo State, Nigeria	6.74	6.22
Ekpoma 2 tibrovirus	2015	No					No					Yes Stremlau et al., 2015	2015	Irrua, Edo State, Nigeria	6.74	6.22
Alagoas vesiculovirus	1967	No					No					No				
Chandipura vesiculovirus	1967	No					No					No				
Cocal vesiculovirus	1964	No					No					No				
Indiana vesiculovirus	1958	Yes Patterson et al., 1958	1958	Beltsville, Prince George's County, Maryland	39.05	--76.90	No					No				
Isfahan vesiculovirus	1977	No					No					No				
Maraba vesiculovirus	1984	No					No					No				
New Jersey vesiculovirus	1950	Yes Hanson et al., 1950	1950	Madison, Wisconsin	43.07	--89.40	No					No				
Piry vesiculovirus	1974	No					No					No				
Barmah Forest virus	1986	No					No					No				
Chikungunya virus	1956	Yes* Centers for Disease Control and Prevention, 2006	2006	Minnesota	46.44	--93.36	Yes Clarke et al., 1967	1967	Southwest Taiwan	23.06	120.59	Yes Ross, 1956	1956	Newala district, Tanzania	--10.64	39.24
Eastern equine encephalitis virus	1938	Yes Howitt, 1938	1938	Southwestern Massachusetts	42.19	--73.09	No					No				
Everglades virus	1970	Yes Ehrenkranz et al., 1970	1970	Homestead, Florida	25.47	--80.48	No					No				
Getah virus	1966	No					Yes Li et al., 1992	1992	Baoting County, Hainan	18.98	109.83	No				
Highlands J virus	2000	Yes Meehan et al., 2000	2000	Florida	27.66	--81.52	No					No				
Madariaga virus	1972	No					No					No				
Mayaro virus	1957	Yes* Tesh et al., 1999	1999	Ohio	40.42	--82.91	No					No				
Mosso das Pedras virus	2013	No					No					No				
Mucambo virus	1965	No					No					No				
Ndumu virus	1961	No					No					Yes Kokernot et al., 1961	1961	Ndumu, Maputaland, KwaZulu-Natal, South Africa	--26.93	32.26
Onyong-nyong virus	1961	No					No					Yes Williams and Woodall, 1961	1961	Entebbe, Uganda	0.05	32.46
Pixuna virus	1991	No					No					No				
Rio Negro virus	1993	No					No					No				
Ross River virus	1972	No					Yes Xu et al., 1999	1999	Hainan	19.16	109.94	No				
Semliki Forest virus	1979	No					No					Yes Mathiot et al., 1990	1990	Bangui, Central Africa	4.36	18.58
Sindbis virus	1955	No					No					Yes Taylor et al., 1955	1955	Cairo, Egypt	30.04	31.24
Tonate virus	1976	No					No					No				
Una virus	1963	No					No					No				
Venezuelan equine encephalitis virus	1943	Yes Casals et al., 1943	1943	New York	40.71	--74.01	No					No				
Western equine encephalitis virus	1938	Yes Howitt, 1938	1938	Fresno, California	36.75	--119.77	No					No				
Whataroa virus	1964	No					No					No				
Rubella virus	1942	Yes Habel, 1942	1942	Washington, D. C.	38.91	--77.04	Yes He et al., 1979	1979	Hangzhou, Zhejiang	29.87	119.33	Yes Selzer, 1963	1963	Cape Town, Western Cape Province, South Africa	--33.90	18.57
Hepatitis delta virus	1977	Yes Rizzetto et al., 1979	1979	New Jersey	40.06	--74.41	Yes Rizzetto et al., 1980	1980	Taipei, Taiwan	24.96	121.51	Yes Crocchiolo et al., 1984	1984	Harare, Zimbabwe	--17.83	31.03

Notes: Yes denotes the virus was ever discovered from the region; * denotes the virus was ever discovered from the region, but imported from other regions; No denotes the virus species has never been discovered from the region; The lat and long denote the coordidate of discovery points or centroids of polygons.

**Appendix 1—table 2.** Resolution and covered grid cells for virus discovery data.

		Polygon data			Point data	Total
		Country level	State/Province level	City/County level		
**United States**	**Virus counts**	NA	14 (14.7%)	11 (11.6%)	70 (73.7%)	95
	**Gridded cell counts**	NA	189	12	72^*^	273
**China**	**Virus counts**	NA	22 (27.5%)	47 (58.7%)	11 (13.8%)	80
	**Gridded cell counts**	NA	161	70	12^*^	243
	**Virus counts**	7 (6.5%)	5 (4.7%)	15 (14.0%)	80 (74.8%)	107
**Africa**	**Gridded cell counts**	307	22	17	80	426

* Grid cell counts here include viruses first detected in multiple points from the literature, NA, not applicable.

**Appendix 1—table 3.** Model parameters.

Model	Tree complexity	Learning rate	Bag fraction	No. of trees
**United States**	2	0.0020	0.5	1430
**China**	2	0.0035	0.5	1473
**Africa**	2	0.0030	0.5	1446

**Appendix 1—table 4.** Model validation statistics for analyses in three regions.

Model	% of deviance explained (95% quantiles)	ICC (95% quantiles)
**United States**	50.5% (44.3%–56.8%)	0.66 (0.60–0.70)
**China**	42.0% (32.4%–50.8%)	0.52 (0.41–0.60)
**Africa**	42.4% (34.2%–50.0%)	0.51 (0.44–0.62)

ICC, intraclass correlation coefficient.

## Appendix 2

We considered using bibliographic data to adjust for discovery effort, but rejected this strategy after some exploratory tests. Jones et al., 2008 estimated the discovery effort for emerging infectious diseases (EID) by calculating the number of papers published by each country (denoted by the address for every author) in the Journal of Infectious Diseases (JID) since 1973. The hypothesis is that countries publishing more papers in JID are likely to discover more EID events. We tested whether this method worked for our analysis by plotting the relationship between published human-infective RNA virus count and total number of papers from all journals which published on human-infective RNA viruses in Web of Science (as of 21 Feb 2018). Both the total number of papers (Appendix 3—figure 1A) and total number of papers on viruses (Appendix 3—figure 1B) were weakly linked to the published human virus count in our database, though the number of papers did have a positive relationship with the number of papers on viruses (Appendix 3—figure 1C). We also noted that papers in JID (highlighted in blue in Appendix 3—figure 1) may not be able to fully explain the discovery efforts for newly discovered viruses. Olival et al., 2017 adjust for the discovery effort by searching the number of publications for each of 586 virus species they have studied using a keyword search by virus name in PubMed and Web of Science. We found the results using this method were similar to that of Jones et al., 2008. Allen et al., 2017 derived a different index for discovery bias, based on the spatial distribution of place names in peer-reviewed biomedical literature. The disadvantage of this method is that it may not represent the discovery effort, as many place names are not related to zoonotic viruses.

## Appendix 4

As covariates may vary within a decade and their effects on virus discovery were likely not immediate, we performed two further sensitivity analyses by (i) matching virus discovery data and time-varying covariate data by year and (ii) testing for lag effects by matching virus discovery at year t and predictors at t-1 to t-5 year. We collected yearly data for climatic variables and land use from the same sources used in the main analysis. Yearly population data at grid level before 1970 and GDP data before 1980 are not available, so we extrapolated them back to 1901 using the yearly growth rate at country level (Source: Our World in Data). For population, the WorldPop Project provides yearly gridded data for 2000-2020 (https://www.arcgis.com/home/item.html?id=56eb0f050c61434782f008a08331d23a), and we used the growth rate by grid to extrapolate values after 2000.

## References

[bib1] Abroug F, Slim A, Ouanes-Besbes L, Hadj Kacem M-A, Dachraoui F, Ouanes I, Lu X, Tao Y, Paden C, Caidi H, Miao C, Al-Hajri MM, Zorraga M, Ghaouar W, BenSalah A, Gerber SI, World Health Organization Global Outbreak Alert and Response Network Middle East Respiratory Syndrome Coronavirus International Investigation Team (2014). Family cluster of Middle East respiratory syndrome coronavirus infections, Tunisia, 2013. Emerging Infectious Diseases.

[bib2] Achong BG, Mansell PW, Epstein MA (1971). A new human virus in cultures from A nasopharyngeal carcinoma. The Journal of Pathology.

[bib3] Albariño CG, Shoemaker T, Khristova ML, Wamala JF, Muyembe JJ, Balinandi S, Tumusiime A, Campbell S, Cannon D, Gibbons A, Bergeron E, Bird B, Dodd K, Spiropoulou C, Erickson BR, Guerrero L, Knust B, Nichol ST, Rollin PE, Ströher U (2013). Genomic analysis of filoviruses associated with four viral hemorrhagic fever outbreaks in Uganda and the Democratic Republic of the Congo in 2012. Virology.

[bib4] Albariño CG, Foltzer M, Towner JS, Rowe LA, Campbell S, Jaramillo CM, Bird BH, Reeder DM, Vodzak ME, Rota P, Metcalfe MG, Spiropoulou CF, Knust B, Vincent JP, Frace MA, Nichol ST, Rollin PE, Ströher U (2014). Novel paramyxovirus associated with severe acute febrile disease, South Sudan and Uganda, 2012. Emerging Infectious Diseases.

[bib5] Allen T, Murray KA, Zambrana-Torrelio C, Morse SS, Rondinini C, Di Marco M, Breit N, Olival KJ, Daszak P (2017). Global hotspots and correlates of emerging zoonotic diseases. Nature Communications.

[bib6] Andayi F, Charrel RN, Kieffer A, Richet H, Pastorino B, Leparc-Goffart I, Ahmed AA, Carrat F, Flahault A, de Lamballerie X (2014). A sero-epidemiological study of arboviral fevers in Djibouti, Horn of Africa. PLOS Neglected Tropical Diseases.

[bib7] Armstrong C, Lillie RD (1934). Experimental Lymphocytic Choriomeningitis of Monkeys and Mice Produced by a Virus Encountered in Studies of the 1933 St Louis Encephalitis Epidemic. Public Health Reports (1896-1970).

[bib8] Baker KS, Todd S, Marsh GA, Crameri G, Barr J, Kamins AO, Peel AJ, Yu M, Hayman DTS, Nadjm B, Mtove G, Amos B, Reyburn H, Nyarko E, Suu-Ire R, Murcia PR, Cunningham AA, Wood JLN, Wang L-F (2013). Novel, potentially zoonotic paramyxoviruses from the African straw-colored fruit bat Eidolon helvum. Journal of Virology.

[bib9] Balestrieri A, Russo V, D’Arrigo C (1967). Serum haemagglutination-inhibiting antibodies for haemadsorbing viruses types 1 and 3 and croup-associated in persons in Accra, Ghana. Arch Ital Sci Med Trop e Parassit.

[bib10] Bardos V, Sefcovicova L (1961). The presence of antibodies neutralizing Tahyna virus in the sera of inhabitants of some European, Asian, African and Australian countries. Journal of Hygiene, Epidemiology, Microbiology, and Immunology.

[bib11] Barry M, Russi M, Armstrong L, Geller D, Tesh R, Dembry L, Gonzalez JP, Khan AS, Peters CJ (1995). Brief report: treatment of a laboratory-acquired Sabiá virus infection. The New England Journal of Medicine.

[bib12] Bayer P, Gear J (1955). Virus meningo-encephalitis in South Africa; a study of the cases admitted to the Johannesburg Fever Hospital. South African Journal of Laboratory and Clinical Medicine. Suid-Afrikaanse Tydskrif Vir Laboratorium- En Kliniekwerk.

[bib13] Baylet R, Schluep R, Cantrelle DS, Rey M (1963). Age-Grouping in Measles in an Urban Environment (A Serological Study. Bulletin de La Societe Medicale d’Afrique Noire de Langue Francaise.

[bib14] Belabbes EH, Bouguermouh A, Benatallah A, Illoul G (1985). Epidemic non-A, non-B viral hepatitis in Algeria: strong evidence for its spreading by water. Journal of Medical Virology.

[bib15] Bialek SR, Allen D, Alvarado-Ramy F, Arthur R, Balajee A, Bell D, Best S, Blackmore C, Breakwell L, Cannons A, Brown C, Cetron M, Chea N, Chommanard C, Cohen N, Conover C, Crespo A, Creviston J, Curns AT, Dahl R, Dearth S, DeMaria A, Echols F, Erdman DD, Feikin D, Frias M, Gerber SI, Gulati R, Hale C, Haynes LM, Heberlein-Larson L, Holton K, Ijaz K, Kapoor M, Kohl K, Kuhar DT, Kumar AM, Kundich M, Lippold S, Liu L, Lovchik JC, Madoff L, Martell S, Matthews S, Moore J, Murray LR, Onofrey S, Pallansch MA, Pesik N, Pham H, Pillai S, Pontones P, Pringle K, Pritchard S, Rasmussen S, Richards S, Sandoval M, Schneider E, Schuchat A, Sheedy K, Sherin K, Swerdlow DL, Tappero JW, Vernon MO, Watkins S, Watson J, Centers for Disease Control and Prevention (CDC) (2014). First confirmed cases of Middle East respiratory syndrome coronavirus (MERS-CoV) infection in the United States, updated information on the epidemiology of MERS-CoV infection, and guidance for the public, clinicians, and public health authorities - May 2014. MMWR. Morbidity and Mortality Weekly Report.

[bib16] Black SP, Powers LM (1910). History of Rabies in Southern California. California State Journal of Medicine.

[bib17] Bode L, Riegel S, Lange W, Ludwig H (1992). Human infections with Borna disease virus: seroprevalence in patients with chronic diseases and healthy individuals. Journal of Medical Virology.

[bib18] Bowen ET, Lloyd G, Harris WJ, Platt GS, Baskerville A, Vella EE (1977). Viral haemorrhagic fever in southern Sudan and northern Zaire: Preliminary studies on the aetiological agent. Lancet (London, England).

[bib19] Briese T, Renwick N, Venter M, Jarman RG, Ghosh D, Köndgen S, Shrestha SK, Hoegh AM, Casas I, Adjogoua EV, Akoua-Koffi C, Myint KS, Williams DT, Chidlow G, van den Berg R, Calvo C, Koch O, Palacios G, Kapoor V, Villari J, Dominguez SR, Holmes KV, Harnett G, Smith D, Mackenzie JS, Ellerbrok H, Schweiger B, Schønning K, Chadha MS, Leendertz FH, Mishra AC, Gibbons RV, Holmes EC, Lipkin WI (2008). Global distribution of novel rhinovirus genotype. Emerging Infectious Diseases.

[bib20] Briese T, Paweska JT, McMullan LK, Hutchison SK, Street C, Palacios G, Khristova ML, Weyer J, Swanepoel R, Egholm M, Nichol ST, Lipkin WI (2009). Genetic detection and characterization of Lujo virus, a new hemorrhagic fever-associated arenavirus from southern Africa. PLOS Pathogens.

[bib21] Brun-Vézinet F, Rouzioux C, Montagnier L, Chamaret S, Gruest J, Barré-Sinoussi F, Geroldi D, Chermann JC, McCormick J, Mitchell S (1984). Prevalence of antibodies to lymphadenopathy-associated retrovirus in African patients with AIDS. Science (New York, N.Y.).

[bib22] Buchmeier M, Adam E, Rawls WE (1974). Serological evidence of infection by Pichinde virus among laboratory workers. Infection and Immunity.

[bib23] Buckley SM, Casals J (1970). Lassa fever, a new virus disease of man from West Africa. 3. Isolation and characterization of the virus. The American Journal of Tropical Medicine and Hygiene.

[bib24] Burnet FM (1943). HUMAN INFECTION WITH THE VIRUS OF NEWCASTLE DISEASE OF FOWLS. Medical Journal of Australia.

[bib25] Calattini S, Chevalier SA, Duprez R, Bassot S, Froment A, Mahieux R, Gessain A (2005). Discovery of a new human T-cell lymphotropic virus (HTLV-3) in Central Africa. Retrovirology.

[bib26] Carroll D, Daszak P, Wolfe ND, Gao GF, Morel CM, Morzaria S, Pablos-Méndez A, Tomori O, Mazet JAK (2018). The Global Virome Project. Science (New York, N.Y.).

[bib27] Casals J, Curnen EC, Thomas L (1943). VENEZUELAN EQUINE ENCEPHALOMYELITIS IN MAN. The Journal of Experimental Medicine.

[bib28] Causey OR, Kemp GE, Madbouly MH, Lee VH (1969). Arbovirus surveillance in Nigeria, 1964-1967. Bulletin de La Societe de Pathologie Exotique et de Ses Filiales.

[bib29] Centers for Disease Control (1988). AIDS due to HIV-2 infection--New Jersey. MMWR. Morbidity and Mortality Weekly Report.

[bib30] Centers for Disease Control and Prevention (2006). Chikungunya fever diagnosed among international travelers--United States, 2005-2006. MMWR. Morbidity and Mortality Weekly Report.

[bib31] Centers for Disease Control and Prevention (2009). Imported case of Marburg hemorrhagic fever - Colorado, 2008. MMWR. Morbidity and Mortality Weekly Report.

[bib32] Chan KH, Cheng VCC, Woo PCY, Lau SKP, Poon LLM, Guan Y, Seto WH, Yuen KY, Peiris JSM (2005). Serological responses in patients with severe acute respiratory syndrome coronavirus infection and cross-reactivity with human coronaviruses 229E, OC43, and NL63. Clinical and Diagnostic Laboratory Immunology.

[bib33] Chang HT, Chiang YT (1950). Studies on an epidemic of influenza in Peking. Chinese Medical Journal.

[bib34] Chang RS, Chan RC, French GL, Leong S, Mak KH, Carlson JR, Yee J, Gardner MB (1986). HTLV-III antibody testing in Hong Kong. JAMA.

[bib35] Chanock RM (1956). Association of a new type of cytopathogenic myxovirus with infantile croup. The Journal of Experimental Medicine.

[bib36] Chanock R, Roizman B, Myers R (1957). RECOVERY FROM INFANTS WITH RESPIRATORY ILLNESS OF A VIRUS RELATED TO CHIMPANZEE CORYZA AGENT (CCA. American Journal of Epidemiology.

[bib37] Chanock RM, Parrott RH, Cook K, Andrews BE, Bell JA, Reichelderfer T, Kapikian AZ, Mastrota FM, Huebner RJ (1958). Newly recognized myxoviruses from children with respiratory disease. The New England Journal of Medicine.

[bib38] Charles M C (2003). Severe acute respiratory syndrome (SARS) and coronavirus testing - United States, 2003 (Reprinted from MMWR, vol 52, pg 297-302, 2003. Jama-J Am Med Assoc.

[bib39] Chen ZH, Zhang EH, Zhang XZ, He NX (1964). Isolation of parainfluenza type I virus by tissue culture and adsorption-hemagglutination test [Article in Chinese]. Journal of Zhejiang University (Medical Sciences).

[bib40] Chen CH, Chiu YL, Wei FC, Koong FJ, Liu HC, Shaw CK, Hwu HG, Hsiao KJ (1999). High seroprevalence of Borna virus infection in schizophrenic patients, family members and mental health workers in Taiwan. Molecular Psychiatry.

[bib41] Chen J, Lu H (2016). Yellow fever in China is still an imported disease. Bioscience Trends.

[bib42] Cheng WX, Cui SX, Jin Y, Duan ZJ (2009a). New Saffold cardiovirus in children, China. Emerging Infectious Diseases.

[bib43] Cheng P, Lau CS, Lai A, Ho E, Leung P, Chan F, Wong A, Lim W (2009b). A novel reovirus isolated from A patient with acute respiratory disease. Journal of Clinical Virology.

[bib44] Chhabra P, Payne DC, Szilagyi PG, Edwards KM, Staat MA, Shirley SH, Wikswo M, Nix WA, Lu X, Parashar UD, Vinjé J (2013). Etiology of viral gastroenteritis in children <5 years of age in the United States, 2008-2009. The Journal of Infectious Diseases.

[bib45] Chiu W-T, Huang J-S, Ho Y-S (2004). Bibliometric analysis of Severe Acute Respiratory Syndrome-related research in the beginning stage. Scientometrics.

[bib46] Choo QL, Kuo G, Weiner AJ, Overby LR, Bradley DW, Houghton M (1989). Isolation of A cDNA clone derived from A blood-borne non-A, non-B viral hepatitis genome. Science (New York, N.Y.).

[bib47] Chu DKW, Chin AWH, Smith GJ, Chan K-H, Guan Y, Peiris JSM, Poon LLM (2010). Detection of novel astroviruses in urban brown rats and previously known astroviruses in humans. The Journal of General Virology.

[bib48] Cicchetti DV (1994). Guidelines, criteria, and rules of thumb for evaluating normed and standardized assessment instruments in psychology. Psychological Assessment.

[bib49] Clarke EJ, Suitor EC, Jenkin HM (1967). A serological survey of arboviruses in the human population of Senegal. Tropical and Geographical Medicine.

[bib50] Cliff AD, Ord JK (1981). Spatial processes: Models and applications. Pion Limited.

[bib51] Crase B, Liedloff AC, Wintle BA (2012). A new method for dealing with residual spatial autocorrelation in species distribution models. Ecography.

[bib52] Crocchiolo PR, Caredda F, D’Arminio Monforte A, Lencioni R, Ragni MC, Cenzuales S, Farci P, Lavarini C, Latif AS (1984). The aetiology of acute hepatitis in Zimbabwe. Transactions of the Royal Society of Tropical Medicine and Hygiene.

[bib53] Cruse RP, Rothner AD, Erenberg G, Calisher CH (1979). Central European Tick-borne Encephalitis: An Ohio Case With a History of Foreign Travel. Archives of Pediatrics & Adolescent Medicine.

[bib54] Dai XQ, Hua XG, Shan TL, Delwart E, Zhao W (2010). Human cosavirus infections in children in China. Journal of Clinical Virology.

[bib55] Daubney R, Hudson JR, Garnham PC (1931). Enzootic hepatitis or rift valley fever: An undescribed virus disease of sheep cattle and man from east africa. The Journal of Pathology and Bacteriology.

[bib56] De Cock KM, Bradley DW, Sandford NL, Govindarajan S, Maynard JE, Redeker AG (1987). Epidemic non-A, non-B hepatitis in patients from Pakistan. Annals of Internal Medicine.

[bib57] Delaporte E, Louwagie J, Peeters M, Montplaisir N, d’Auriol L, Ville Y, Bedjabaga L, Larouzé B, Van der Groen G, Piot P (1991). Evidence of HTLV-II infection in central Africa. AIDS (London, England).

[bib58] Dick GWA, Best AM (1948). Mengo encephalomyelitis; a hitherto unknown virus affecting man. Lancet (London, England).

[bib59] Dick GW (1952). Zika virus. II. Pathogenicity and Physical Properties. Transactions of the Royal Society of Tropical Medicine and Hygiene.

[bib60] Dick GW, Haddow AJ (1952). Uganda S virus. A hitherto unrecorded virus isolated from mosquitoes in Uganda. I. Isolation and Pathogenicity. Transactions of the Royal Society of Tropical Medicine and Hygiene.

[bib61] Doggett JE (1965). Antibodies to respiratory syncytial virus in human sera from different regions of the world. Bulletin of the World Health Organization.

[bib62] Donia HA, Youssef BZ (2002). Foot and mouth disease (FMD): serological investigation in some farms of Alexandria Governorate of Egypt. The Journal of the Egyptian Public Health Association.

[bib63] Dowling JM, Wynne H (1981). Role of enteric adenoviruses and rotaviruses in infantile gastroenteritis. Lancet (London, England).

[bib64] Edington AD (1927). "Dengue " as seen in the Recent Epidemic in Durban. J Med Assoc S Africa.

[bib65] Ehrenkranz NJ, Sinclair MC, Buff E, Lyman DO (1970). The natural occurrence of Venezuelan equine encephalitis in the United States. The New England Journal of Medicine.

[bib66] Eiden J, Vonderfecht S, Yolken RH (1985). Evidence that a novel rotavirus-like agent of rats can cause gastroenteritis in man. Lancet (London, England).

[bib67] Elith J, Leathwick JR, Hastie T (2008). A working guide to boosted regression trees. The Journal of Animal Ecology.

[bib68] Enserink M (2000). Emerging diseases: New arenavirus blamed for recent deaths in California. Science (New York, N.Y.).

[bib69] Esper F, Weibel C, Ferguson D, Landry ML, Kahn JS (2005). Evidence of a novel human coronavirus that is associated with respiratory tract disease in infants and young children. The Journal of Infectious Diseases.

[bib70] Esper F, Weibel C, Ferguson D, Landry ML, Kahn JS (2006). Coronavirus HKU1 infection in the United States. Emerging Infectious Diseases.

[bib71] Falsey AR, Erdman D, Anderson LJ, Walsh EE (2003). Human metapneumovirus infections in young and elderly adults. The Journal of Infectious Diseases.

[bib72] Familusi JB, Osunkoya BO, Moore DL, Kemp GE, Fabiyi A (1972). A fatal human infection with Mokola virus. The American Journal of Tropical Medicine and Hygiene.

[bib73] Fang ZY, Wen LY, Jin SJ, Zhao ZH (1995). Norwalk-like virus infection found in diarrhea patients in China [Article in Chinese. Chinese Journal of Virology.

[bib74] Feinstone SM, Kapikian AZ, Purceli RH (1973). Hepatitis A: detection by immune electron microscopy of A viruslike antigen associated with acute illness. Science (New York, N.Y.).

[bib75] Feng R, Wei J, Zhang H, Fan J, Li X, Wang D, Xie J, Qiao Z, Li M, Bai J, Ma Z (2015). National serosurvey of encephalomyocarditis virus in healthy people and pigs in China. Archives of Virology.

[bib76] Finkbeiner SR, Holtz LR, Jiang Y, Rajendran P, Franz CJ, Zhao G, Kang G, Wang D (2009a). Human stool contains a previously unrecognized diversity of novel astroviruses. Virology Journal.

[bib77] Finkbeiner SR, Li Y, Ruone S, Conrardy C, Gregoricus N, Toney D, Virgin HW, Anderson LJ, Vinjé J, Wang D, Tong S (2009b). Identification of a novel astrovirus (astrovirus VA1) associated with an outbreak of acute gastroenteritis. Journal of Virology.

[bib78] Finkbeiner SR, Le BM, Holtz LR, Storch GA, Wang D (2009c). Detection of newly described astrovirus MLB1 in stool samples from children. Emerging Infectious Diseases.

[bib79] Flexner S, Lewis PA (1909). THE TRANSMISSION OF ACUTE POLIOMYELITIS TO MONKEYS. Journal of the American Medical Association.

[bib80] Florio L, Stewart MO, Mugrage ER (1946). The etiology of Colorado tick fever. The Journal of Experimental Medicine.

[bib81] Forthal DN, Bauer SP, McCormick JB (1987). Antibody to hemorrhagic fever with renal syndrome viruses (Hantaviruses) in the United States. American Journal of Epidemiology.

[bib82] Foy BD, Kobylinski KC, Chilson Foy JL, Blitvich BJ, Travassos da Rosa A, Haddow AD, Lanciotti RS, Tesh RB (2011). Probable non-vector-borne transmission of Zika virus, Colorado, USA. Emerging Infectious Diseases.

[bib83] Francis T, Magill TP (1935). IMMUNOLOGICAL STUDIES WITH THE VIRUS OF INFLUENZA. The Journal of Experimental Medicine.

[bib84] Francis T (1940). Differentiation of Influenza A and Influenza B by the Complement-Fixation Reaction. Experimental Biology and Medicine.

[bib85] Francis T, Quilligan JJ, Minuse E (1950). Identification of another epidemic respiratory disease. Science (New York, N.Y.).

[bib86] Gao J, Song P (2015). China upgrades surveillance and control measures of Middle East respiratory syndrome (MERS). Bioscience Trends.

[bib87] Gear JS, Cassel GA, Gear AJ, Trappler B, Clausen L, Meyers AM, Kew MC, Bothwell TH, Sher R, Miller GB, Schneider J, Koornhof HJ, Gomperts ED, Isaäcson M, Gear JH (1975). Outbreake of Marburg virus disease in Johannesburg. British Medical Journal.

[bib88] Georges AJ, Gonzalez JP, Abdul-Wahid S, Saluzzo JF, Meunier DM, McCormick JB (1985). Antibodies to Lassa and Lassa-like viruses in man and mammals in the Central African Republic. Transactions of the Royal Society of Tropical Medicine and Hygiene.

[bib89] Giangaspero M, Wellemans G, Vanopdenbosch E, Belloli A, Verhulst A (1988). Bovine viral diarrhoea. Lancet (London, England).

[bib90] Goldberger J, Anderson JF (1911). THE NATURE OF THE VIRUS OF MEASLES. Journal of the American Medical Association.

[bib91] Goldfield M, Austin SM, Black HC, Taylor BF, Altman R (1973). A non-fatal human case of Powassan virus encephalitis. The American Journal of Tropical Medicine and Hygiene.

[bib92] Grard G, Fair JN, Lee D, Slikas E, Steffen I, Muyembe J-J, Sittler T, Veeraraghavan N, Ruby JG, Wang C, Makuwa M, Mulembakani P, Tesh RB, Mazet J, Rimoin AW, Taylor T, Schneider BS, Simmons G, Delwart E, Wolfe ND, Chiu CY, Leroy EM (2012). A novel rhabdovirus associated with acute hemorrhagic fever in central Africa. PLOS Pathogens.

[bib93] Greninger AL, Runckel C, Chiu CY, Haggerty T, Parsonnet J, Ganem D, DeRisi JL (2009). The complete genome of klassevirus - a novel picornavirus in pediatric stool. Virology Journal.

[bib94] Grohmann GS, Glass RI, Pereira HG, Monroe SS, Hightower AW, Weber R, Bryan RT (1993). Enteric viruses and diarrhea in HIV-infected patients: Enteric Opportunistic Infections Working Group. The New England Journal of Medicine.

[bib95] Gu HX, Spence L, Artsob H, Chia WK, Th’ng C, Lampotang V (1984). Serological evidence of infection with California serogroup viruses (family Bunyaviridae) in residents of Long Hua, suburb of Shanghai, People’s Republic of China. Transactions of the Royal Society of Tropical Medicine and Hygiene.

[bib96] Guangzhou Institute of Medicine and Health (1975). Investigation report on virus types in patients with cold in Guangzhou [Article in Chinese. Guangdong Medical Journal.

[bib97] Guiteras GM (1904). The Yellow Fever Epidemic of 1903 at Laredo, Texas. JAMA.

[bib98] Habel K (1942). Transmission of Rubella to Macacus mulatta Monkeys. Public Health Reports (1896-1970).

[bib99] Hammon WM, Reeves WC (1952). California encephalitis virus, a newly described agent. California Medicine.

[bib100] Hamre D, Procknow JJ (1961). Viruses isolated from natural common colds in the U.S.A. British Medical Journal.

[bib101] Hamre D, Procknow JJ (1966). A New Virus Isolated from the Human Respiratory Tract. Experimental Biology and Medicine.

[bib102] Hanson RP, Rasmussen AF, Brandly CA, Brown JW (1950). Human infection with the virus of vesicular stomatitis. The Journal of Laboratory and Clinical Medicine.

[bib103] Hassell JM, Begon M, Ward MJ, Fèvre EM (2017). Urbanization and Disease Emergence: Dynamics at the Wildlife-Livestock-Human Interface. Trends in Ecology & Evolution.

[bib104] Hays JP, Myint SH (1998). PCR sequencing of the spike genes of geographically and chronologically distinct human coronaviruses 229E. Journal of Virological Methods.

[bib105] He NX, Xu TZ, Ma JY, Wang XZ, Wang L, Guo MF, Pan CM (1979). Isolation of rubella virus [Article in Chinese. Journal of Zhejiang University (Medical Sciences).

[bib106] Howitt B (1938). Recovery of the Virus of Equine Encephalomyelitis from the Brain of a Child. Science (New York, N.Y.).

[bib107] Huang RT, Wei J, Tian X, Li DR, Yin SR (1989). Isolation of A small RNA virus from feces of A patient with enterically transmitted Non-A Non-B hepatitis in China [Article in Chinese. Journal of Academy of Military Medical Sciences.

[bib108] Huang F, Wang H, Jing S, Zeng W (2012). Simian foamy virus prevalence in Macaca mulatta and zookeepers. AIDS Research and Human Retroviruses.

[bib109] Hudson NP, Lennette EH (1933). THE NEUTRALIZATION OF POLIOMYELITIS VIRUS BY THE SERUM OF LIBERIAN NEGROES*. American Journal of Epidemiology.

[bib110] Hung T, Chen GM, Wang CG, Yao HL, Fang ZY, Chao TX, Chou ZY, Ye W, Chang XJ, Den SS (1984). Waterborne outbreak of rotavirus diarrhoea in adults in China caused by a novel rotavirus. Lancet (London, England).

[bib111] International Committee on Taxonomy of Viruses (2018). Virus Taxonomy. https://talk.ictvonline.org/ictv-reports/ictv_online_report/.

[bib112] Isaacs A, Andrews CH (1951). The spread of influenza; evidence from 1950-1951. British Medical Journal.

[bib113] Jiang B, Dennehy PH, Spangenberger S, Gentsch JR, Glass RI (1995). First detection of group C rotavirus in fecal specimens of children with diarrhea in the United States. The Journal of Infectious Diseases.

[bib114] Johnson CD, Goodpasture EW (1934). AN INVESTIGATION OF THE ETIOLOGY OF MUMPS. The Journal of Experimental Medicine.

[bib115] Johnson KM, Chanock RM, Cook MK, Huebner RJ, Chi L, Wong D, Turner HC (1960). STUDIES OF A NEW HUMAN HEMADSORPTION VIRUS. American Journal of Epidemiology.

[bib116] Johnson KM, Lange JV, Webb PA, Murphy FA (1977). Isolation and partial characterisation of a new virus causing acute haemorrhagic fever in Zaire. Lancet (London, England).

[bib117] Jones MS, Lukashov VV, Ganac RD, Schnurr DP (2007). Discovery of a novel human picornavirus in a stool sample from a pediatric patient presenting with fever of unknown origin. Journal of Clinical Microbiology.

[bib118] Jones KE, Patel NG, Levy MA, Storeygard A, Balk D, Gittleman JL, Daszak P (2008). Global trends in emerging infectious diseases. Nature.

[bib119] Jonkers AH (1961). Serosurvey of encephalomyocarditis virus neutralizing antibodies in southern Louisiana and Peruvian Indian populations. The American Journal of Tropical Medicine and Hygiene.

[bib120] Joosting AC, Head B, Bynoe ML, Tyrrell DA (1968). Production of common colds in human volunteers by influenza C virus. British Medical Journal.

[bib121] Kalyanaraman VS, Sarngadharan MG, Robert-Guroff M, Miyoshi I, Golde D, Gallo RC (1982). A new subtype of human T-cell leukemia virus (HTLV-II) associated with A T-cell variant of hairy cell leukemia. Science (New York, N.Y.).

[bib122] Kanki PJ, Barin F, M’Boup S, Allan JS, Romet-Lemonne JL, Marlink R, McLane MF, Lee TH, Arbeille B, Denis F (1986). New human T-lymphotropic retrovirus related to simian T-lymphotropic virus type III (STLV-IIIAGM. Science (New York, N.Y.).

[bib123] Kapikian AZ, Wyatt RG, Dolin R, Thornhill TS, Kalica AR, Chanock RM (1972). Visualization by immune electron microscopy of a 27-nm particle associated with acute infectious nonbacterial gastroenteritis. Journal of Virology.

[bib124] Kapikian AZ, Kim HW, Wyatt RG (1976). Recent advances in the aetiology of viral gastroenteritis. Ciba Foundation Symposium.

[bib125] Kapoor A, Li L, Victoria J, Oderinde B, Mason C, Pandey P, Zaidi SZ, Delwart E (2009). Multiple novel astrovirus species in human stool. The Journal of General Virology.

[bib126] Kapoor A, Kumar A, Simmonds P, Bhuva N, Singh Chauhan L, Lee B, Sall AA, Jin Z, Morse SS, Shaz B, Burbelo PD, Lipkin WI (2015). Virome Analysis of Transfusion Recipients Reveals a Novel Human Virus That Shares Genomic Features with Hepaciviruses and Pegiviruses. MBio.

[bib127] Kapusinszky B, Phan TG, Kapoor A, Delwart E (2012). Genetic diversity of the genus Cosavirus in the family Picornaviridae: a new species, recombination, and 26 new genotypes. PLOS ONE.

[bib128] Karzon DT (1955). Studies on a neutralizing antibody against canine distemper virus found in man. Pediatrics.

[bib129] Kayali G, Ortiz EJ, Chorazy ML, Nagaraja KV, DeBeauchamp J, Webby RJ, Gray GC (2011). Serologic evidence of avian metapneumovirus infection among adults occupationally exposed to Turkeys. Vector Borne and Zoonotic Diseases (Larchmont, N.Y.).

[bib130] Kew MC, Houghton M, Choo QL, Kuo G (1990). Hepatitis C virus antibodies in southern African blacks with hepatocellular carcinoma. Lancet (London, England).

[bib131] Khabbaz RF, Rowe T, Murphey-Corb M, Heneine WM, Schable CA, George JR, Pau CP, Parekh BS, Lairmore MD, Curran JW (1992). Simian immunodeficiency virus needlestick accident in a laboratory worker. Lancet (London, England).

[bib132] Klempa B, Koivogui L, Sylla O, Koulemou K, Auste B, Krüger DH, ter Meulen J (2010). Serological evidence of human hantavirus infections in Guinea, West Africa. The Journal of Infectious Diseases.

[bib133] Kokernot RH, Mcintosh BM, Worth CB (1961). Ndumu virus, a hitherto unknown agent, isolated from culicine mosouitoes collected in northern Natal: Union of South Africa. The American Journal of Tropical Medicine and Hygiene.

[bib134] Kreuder Johnson C, Hitchens PL, Smiley Evans T, Goldstein T, Thomas K, Clements A, Joly DO, Wolfe ND, Daszak P, Karesh WB, Mazet JK (2015). Spillover and pandemic properties of zoonotic viruses with high host plasticity. Scientific Reports.

[bib135] Ksiazek TG, Erdman D, Goldsmith CS, Zaki SR, Peret T, Emery S, Tong S, Urbani C, Comer JA, Lim W, Rollin PE, Dowell SF, Ling A-E, Humphrey CD, Shieh W-J, Guarner J, Paddock CD, Rota P, Fields B, DeRisi J, Yang J-Y, Cox N, Hughes JM, LeDuc JW, Bellini WJ, Anderson LJ, SARS Working Group (2003). A novel coronavirus associated with severe acute respiratory syndrome. The New England Journal of Medicine.

[bib136] Kun Number 323 Unit, the Chinese People’s Liberation Army (1975). Studies on the isolation and growth characteristics of respiratory syncytial virus [Article in Chinese. Acta Microbiologica Sinica.

[bib137] Lamson D, Renwick N, Kapoor V, Liu Z, Palacios G, Ju J, Dean A, St George K, Briese T, Lipkin WI (2006). MassTag polymerase-chain-reaction detection of respiratory pathogens, including a new rhinovirus genotype, that caused influenza-like illness in New York State during 2004-2005. The Journal of Infectious Diseases.

[bib138] Lau SKP, To W-K, Tse PWT, Chan AKH, Woo PCY, Tsoi H-W, Leung AFY, Li KSM, Chan PKS, Lim WWL, Yung RWH, Chan K-H, Yuen K-Y (2005). Human parainfluenza virus 4 outbreak and the role of diagnostic tests. Journal of Clinical Microbiology.

[bib139] Lau SKP, Yip CCY, Tsoi H-W, Lee RA, So L-Y, Lau Y-L, Chan K-H, Woo PCY, Yuen K-Y (2007). Clinical features and complete genome characterization of a distinct human rhinovirus (HRV) genetic cluster, probably representing a previously undetected HRV species, HRV-C, associated with acute respiratory illness in children. Journal of Clinical Microbiology.

[bib140] Lavinder CH, Francis E (1914). The Etiology of Dengue: An Attempt to Produce the Disease in the Rhesus Monkey by the Inoculation of Defibrinated Blood. Journal of Infectious Diseases.

[bib141] Le Guenno B, Formenty P, Formentry P, Wyers M, Gounon P, Walker F, Boesch C (1995). Isolation and partial characterisation of a new strain of Ebola virus. Lancet (London, England).

[bib142] Lee PW, Gajdusek DC, Gibbs CJ (1980). Aetiological relation between Korean haemorrhagic fever with renal syndrome in People’s Republic of China. Lancet (London, England).

[bib143] Li XD, Qiu FX, Yang H, Rao YN, Calisher CH (1992). Isolation of Getah virus from mosquitos collected on Hainan Island, China, and results of a serosurvey. The Southeast Asian Journal of Tropical Medicine and Public Health.

[bib144] Li L, Victoria J, Kapoor A, Blinkova O, Wang C, Babrzadeh F, Mason CJ, Pandey P, Triki H, Bahri O, Oderinde BS, Baba MM, Bukbuk DN, Besser JM, Bartkus JM, Delwart EL (2009). A novel picornavirus associated with gastroenteritis. Journal of Virology.

[bib145] Li X-L, Fu S-H, Liu W-B, Wang H-Y, Lu Z, Tong S-X, Li Z-X, Nasci RS, Kosoy O, Cui Y, Liang G-D (2013). West nile virus infection in Xinjiang, China. Vector Borne and Zoonotic Diseases (Larchmont, N.Y.).

[bib146] Li LM, Grassly NC, Fraser C (2014). Genomic analysis of emerging pathogens: methods, application and future trends. Genome Biology.

[bib147] Lipkin WI, Firth C (2013). Viral surveillance and discovery. Current Opinion in Virology.

[bib148] Liu Y, Zhang S, Zhao J, Zhang F, Hu R (2013). Isolation of Irkut virus from a Murina leucogaster bat in China. PLOS Neglected Tropical Diseases.

[bib149] Liu W, Sun FJ, Tong YG, Zhang SQ, Cao WC (2016). Rift Valley fever virus imported into China from Angola. The Lancet Infectious Diseases.

[bib150] Luo RH, Xie JQ, Chen YM, Yang SJ (1999). Report of one case of hand-foot-mouth disease in human [Article in Chinese. New Medicine.

[bib151] Ma Y, Zheng S, Wang N, Duan Y, Sun X, Jin J, Zang W, Li M, Wang Y, Zhao G (2013). Epidemiological analysis of HTLV-1 and HTLV-2 infection among different population in Central China. PLOS ONE.

[bib152] Mackay IM, Arden KE (2015). MERS coronavirus: diagnostics, epidemiology and transmission. Virology Journal.

[bib153] Madhi SA, Ludewick H, Abed Y, Klugman KP, Boivin G (2003). Human metapneumovirus-associated lower respiratory tract infections among hospitalized human immunodeficiency virus type 1 (HIV-1)-infected and HIV-1-uninfected African infants. Clinical Infectious Diseases.

[bib154] Malherbe H, Roux P, Kahn E (1963). The Role of Enteropathogenic Bacteria and Viruses in Acute Diarrhoeal Disorders of Infancy and Childhood in Johannesburg. II. “Non-Specific” Gastro-Enteritis. South African Medical Journal = Suid-Afrikaanse Tydskrif Vir Geneeskunde.

[bib155] Mansfield KL, Jizhou L, Phipps LP, Johnson N (2017). Emerging Tick-Borne Viruses in the Twenty-First Century. Frontiers in Cellular and Infection Microbiology.

[bib156] Mathiot CC, Grimaud G, Garry P, Bouquety JC, Mada A, Daguisy AM, Georges AJ (1990). An outbreak of human Semliki Forest virus infections in Central African Republic. The American Journal of Tropical Medicine and Hygiene.

[bib157] McIntosh K, Dees JH, Becker WB, Kapikian AZ, Chanock RM (1967). Recovery in tracheal organ cultures of novel viruses from patients with respiratory disease. PNAS.

[bib158] McMullan LK, Folk SM, Kelly AJ, MacNeil A, Goldsmith CS, Metcalfe MG, Batten BC, Albariño CG, Zaki SR, Rollin PE, Nicholson WL, Nichol ST (2012). A new phlebovirus associated with severe febrile illness in Missouri. The New England Journal of Medicine.

[bib159] Meehan PJ, Wells DL, Paul W, Buff E, Lewis A, Muth D, Hopkins R, Karabatsos N, Tsai TF (2000). Epidemiological features of and public health response to a St Louis encephalitis epidemic in Florida, 1990-1. Epidemiology and Infection.

[bib160] Meredith CD, Prossouw AP, Koch HVP (1971). An unusual case of human rabies thought to be of chiropteran origin. South African Medical Journal = Suid-Afrikaanse Tydskrif Vir Geneeskunde.

[bib161] Microbiology Research Group of Shanghai First Medical College and Laboratory of Shanghai Sixth People’s Hospital (1978). Preliminary report on the examination of hepatitis A antigen particles by immunoelectron microscopy [Article in Chinese. Shanghai Medical Journal.

[bib162] Miranda ME, White ME, Dayrit MM, Hayes CG, Ksiazek TG, Burans JP (1991). Seroepidemiological study of filovirus related to Ebola in the Philippines. Lancet (London, England).

[bib163] Mirkovic RR, Kono R, Yin-Murphy M, Sohier R, Schmidt NJ, Melnick JL (1973). Enterovirus type 70: the etiologic agent of pandemic acute haemorrhagic conjunctivitis. Bulletin of the World Health Organization.

[bib164] Montefiore D, Drozdov SG, Kafuko GW, Fayinka OA, Soneji A (1970). Influenza in East Africa, 1969-70. Bulletin of the World Health Organization.

[bib165] Moore DL, Causey OR, Carey DE, Reddy S, Cooke AR, Akinkugbe FM, David-West TS, Kemp GE (1975). Arthropod-borne viral infections of man in Nigeria, 1964-1970. Annals of Tropical Medicine and Parasitology.

[bib166] Morrill JC, Johnson BK, Hyams C, Okoth F, Tukei PM, Mugambi M, Woody J (1991). Serological evidence of arboviral infections among humans of coastal Kenya. The Journal of Tropical Medicine and Hygiene.

[bib167] Morse SS (2012). Prediction and prevention of the next pandemic zoonosis. Lancet (London, England).

[bib168] Morzunov SP, Feldmann H, Spiropoulou CF, Semenova VA, Rollin PE, Ksiazek TG, Peters CJ, Nichol ST (1995). A newly recognized virus associated with A fatal case of hantavirus pulmonary syndrome in Louisiana. Journal of Virology.

[bib169] Moscovivci C, Laplaca M, Maisel J, Kempe H (1961). Studies of bovine enteroviruses. American Journal of Veterinary Research.

[bib170] Nakata S, Estes MK, Graham DY, Wang SS, Gary GW, Melnick JL (1987). Detection of antibody to group B adult diarrhea rotaviruses in humans. Journal of Clinical Microbiology.

[bib171] Nakata S, Estes MK, Chiba S (1988). Detection of human calicivirus antigen and antibody by enzyme-linked immunosorbent assays. Journal of Clinical Microbiology.

[bib172] Nash D, Mostashari F, Fine A, Miller J, O’Leary D, Murray K, Huang A, Rosenberg A, Greenberg A, Sherman M, Wong S, Layton M, 1999 West Nile Outbreak Response Working Group (2001). The outbreak of West Nile virus infection in the New York City area in 1999. The New England Journal of Medicine.

[bib173] Niang MN, Diop OM, Sarr FD, Goudiaby D, Malou-Sompy H, Ndiaye K, Vabret A, Baril L (2010). Viral etiology of respiratory infections in children under 5 years old living in tropical rural areas of Senegal: The EVIRA project. Journal of Medical Virology.

[bib174] Nichol ST, Spiropoulou CF, Morzunov S, Rollin PE, Ksiazek TG, Feldmann H, Sanchez A, Childs J, Zaki S, Peters CJ (1993). Genetic identification of a hantavirus associated with an outbreak of acute respiratory illness. Science (New York, N.Y.).

[bib175] Olival KJ, Hosseini PR, Zambrana-Torrelio C, Ross N, Bogich TL, Daszak P (2017). Host and viral traits predict zoonotic spillover from mammals. Nature.

[bib176] Oshiro LS, Haley CE, Roberto RR, Riggs JL, Croughan M, Greenberg H, Kapikian A (1981). A 27-nm virus isolated during an outbreak of acute infectious nonbacterial gastroenteritis in A convalescent hospital: A possible new serotype. The Journal of Infectious Diseases.

[bib177] PaPa QF, Qiu FX, Yu FR, Chen SZ (1979). Rotavirus-the source of acute gastroenteritis in infants in autumn [Article in Chinese. Bulletin of Medical Research.

[bib178] Parrish CR, Holmes EC, Morens DM, Park EC, Burke DS, Calisher CH, Laughlin CA, Saif LJ, Daszak P (2008). Cross-species virus transmission and the emergence of new epidemic diseases. Microbiology and Molecular Biology Reviews.

[bib179] Pathogen biology research group, Jiangsu new medical college (1975). Pathogen biology research group Jnmc: Virus isolation in 535 elderly patients with chronic bronchitis and other respiratory infections and antibody tests in some cases [Article in Chinese. Jiangsu Medical Journal.

[bib180] Patterson WC, Mott LO, Jenney EW (1958). A study of vesicular stomatitis in man. Journal of the American Veterinary Medical Association.

[bib181] Patz IM, Measroch V, Gear J (1953). Bornholm disease, pleurodynia or epidemic myalgia; an outbreak in the Transvaal associated with Coxsackie virus infection. South African Medical Journal = Suid-Afrikaanse Tydskrif Vir Geneeskunde.

[bib182] Peiris JSM, Lai ST, Poon LLM, Guan Y, Yam LYC, Lim W, Nicholls J, Yee WKS, Yan WW, Cheung MT, Cheng VCC, Chan KH, Tsang DNC, Yung RWH, Ng TK, Yuen KY, SARS study group (2003a). Coronavirus as a possible cause of severe acute respiratory syndrome. Lancet (London, England).

[bib183] Peiris JSM, Tang WH, Chan KH, Khong PL, Guan Y, Lau YL, Chiu SS (2003b). Children with respiratory disease associated with metapneumovirus in Hong Kong. Emerging Infectious Diseases.

[bib184] Perex-Pina F, Merikangas UR (1953). Japanese B encephalitis in an American soldier returning from Korea. The New England Journal of Medicine.

[bib185] Petti CA, Polage CR, Quinn TC, Ronald AR, Sande MA (2006). Laboratory medicine in Africa: A barrier to effective health care. Clinical Infectious Diseases.

[bib186] Poiesz BJ, Ruscetti FW, Gazdar AF, Bunn PA, Minna JD, Gallo RC (1980). Detection and isolation of type C retrovirus particles from fresh and cultured lymphocytes of a patient with cutaneous T-cell lymphoma. PNAS.

[bib187] Price WH (1956). THE ISOLATION OF A NEW VIRUS ASSOCIATED WITH RESPIRATORY CLINICAL DISEASE IN HUMANS. PNAS.

[bib188] Pulliam JRC, Dushoff J (2009). Ability to replicate in the cytoplasm predicts zoonotic transmission of livestock viruses. The Journal of Infectious Diseases.

[bib189] Qiao H, Nilsson M, Abreu ER, Hedlund KO, Johansen K, Zaori G, Svensson L (1999). Viral diarrhea in children in Beijing, China. Journal of Medical Virology.

[bib190] Rabaa MA (2015). A Strategic Approach to Studying Emerging Zoonotic Infectious Diseases: Ecohealth. The Vietnam Initiative on Zoonotic Infections (VIZIONS.

[bib191] Ramos-Alvarez M, Sabin AB (1954). Characteristics of Poliomyelitis and Other Enteric Viruses Recovered in Tissue Culture from Healthy American Children. Experimental Biology and Medicine.

[bib192] Ramoz-alverz M, Sabin AB (1958). Enteropathogenic viruses and bacteria; role in summer diarrheal diseases of infancy and early childhood. Journal of the American Medical Association.

[bib193] Ravkov EV, Rollin PE, Ksiazek TG, Peters CJ, Nichol ST (1995). Genetic and serologic analysis of Black Creek Canal virus and its association with human disease and Sigmodon hispidus infection. Virology.

[bib194] Ritchie H (2018). Urbanization. https://ourworldindata.org/urbanization.

[bib195] Rivers TM, Schwentker FF (1934). Louping Ill in Man. The Journal of Experimental Medicine.

[bib196] Rizzetto M, Shih JW, Gocke DJ, Purcell RH, Verme G, Gerin JL (1979). Incidence and significance of antibodies to delta antigen in hepatitis B virus infection. Lancet (London, England).

[bib197] Rizzetto M, Purcell RH, Gerin JL (1980). Epidemiology of HBV-associated delta agent: geographical distribution of anti-delta and prevalence in polytransfused HBsAg carriers. Lancet (London, England).

[bib198] Rodgers MA, Holzmayer V, Vallari A, Olivo A, Forberg K, Fuhrman J, Coller KE, Awazi B, Kenmegne Sidje JB, Frankel MB, Berg MG, Mbanya D, Ndembi N, Cloherty GA (2019). Hepatitis C virus surveillance and identification of human pegivirus 2 in a large Cameroonian cohort. Journal of Viral Hepatitis.

[bib199] Rosen BI, Fang ZY, Glass RI, Monroe SS (2000). Cloning of human picobirnavirus genomic segments and development of an RT-PCR detection assay. Virology.

[bib200] Rosenberg R, Johansson MA, Powers AM, Miller BR (2013). Search strategy has influenced the discovery rate of human viruses. PNAS.

[bib201] Rosenberg R (2015). Detecting the emergence of novel, zoonotic viruses pathogenic to humans. Cellular and Molecular Life Sciences.

[bib202] Roser M (2013). Economic Growth. https://ourworldindata.org/economic-growth.

[bib203] Ross RW (1956). The Newala epidemic. III. The Virus: Isolation, Pathogenic Properties and Relationship to the Epidemic. The Journal of Hygiene.

[bib204] Rott R, Herzog S, Fleischer B, Winokur A, Amsterdam J, Dyson W, Koprowski H (1985). Detection of serum antibodies to Borna disease virus in patients with psychiatric disorders. Science (New York, N.Y.).

[bib205] Rua R, Betsem E, Calattini S, Saib A, Gessain A (2012). Genetic characterization of simian foamy viruses infecting humans. Journal of Virology.

[bib206] Sabin AB (1951). Experimental studies on Phlebotomus (pappataci, sandfly) fever during World War II. Archiv Fur Die Gesamte Virusforschung.

[bib207] Safai B, Sarngadharan MG, Groopman JE, Arnett K, Popovic M, Sliski A, Schüpbach J, Gallo RC (1984). Seroepidemiological studies of human T-lymphotropic retrovirus type III in acquired immunodeficiency syndrome. Lancet (London, England).

[bib208] Savage HM, Godsey MS, Lambert A, Panella NA, Burkhalter KL, Harmon JR, Lash RR, Ashley DC, Nicholson WL (2013). First detection of heartland virus (Bunyaviridae: Phlebovirus) from field collected arthropods. The American Journal of Tropical Medicine and Hygiene.

[bib209] Schieble JH, Fox VL, Lennette EH (1967). A probable new human picornavirus associated with respiratory diseases. American Journal of Epidemiology.

[bib210] Schultz EW, Habel K (1959). SA virus; a new member of the myxovirus group. Journal of Immunology (Baltimore, Md.

[bib211] Sdiri-Loulizi K, Gharbi-Khélifi H, de Rougemont A, Chouchane S, Sakly N, Ambert-Balay K, Hassine M, Guédiche MN, Aouni M, Pothier P (2008). Acute infantile gastroenteritis associated with human enteric viruses in Tunisia. Journal of Clinical Microbiology.

[bib212] Sebata T, Steele AD (1999). Human group C rotavirus identified in South Africa. South African Medical Journal = Suid-Afrikaanse Tydskrif Vir Geneeskunde.

[bib213] Selzer G (1963). VIRUS ISOLATION, INCLUSION BODIES, AND CHROMOSOMES IN A RUBELLA-INFECTED HUMAN EMBRYO. Lancet (London, England).

[bib214] Shan TL, Guo W, Cui L, Shang XG, Dai XQ, Yuan CL, Yu Y, Zhang W, Zhu JG, Shen Q, Yang ZB, Hua XG (2009). The first detection of human parechovirus infections in China. Journal of Clinical Virology.

[bib215] Shan T, Wang C, Cui L, Yu Y, Delwart E, Zhao W, Zhu C, Lan D, Dai X, Hua X (2010). Picornavirus salivirus/klassevirus in children with diarrhea, China. Emerging Infectious Diseases.

[bib216] Shanghai Eye and Skin Disease Prevention and Treatment Institute (1979). Isolation and identification of acute hemorrhagic conjunctivitis virus in 1975 [Article in Chinese. Chinese Journal of Ophthalmology.

[bib217] Shearer FM, Longbottom J, Browne AJ, Pigott DM, Brady OJ, Kraemer MUG, Marinho F, Yactayo S, de Araújo VEM, da Nóbrega AA, Fullman N, Ray SE, Mosser JF, Stanaway JD, Lim SS, Reiner RC, Moyes CL, Hay SI, Golding N (2018). Existing and potential infection risk zones of yellow fever worldwide: a modelling analysis. The Lancet Global Health.

[bib218] Sickles GM, Dalldorf G (1949). Serologic differences among strains of the Coxsackie group of viruses. Proceedings of the Society for Experimental Biology and Medicine. Society for Experimental Biology and Medicine (New York, N.Y.).

[bib219] Simon-Loriere E, Faye O, Prot M, Casademont I, Fall G, Fernandez-Garcia MD, Diagne MM, Kipela J-M, Fall IS, Holmes EC, Sakuntabhai A, Sall AA (2017). Autochthonous Japanese Encephalitis with Yellow Fever Coinfection in Africa. The New England Journal of Medicine.

[bib220] Simons JN, Leary TP, Dawson GJ, Pilot-Matias TJ, Muerhoff AS, Schlauder GG, Desai SM, Mushahwar IK (1995). Isolation of novel virus-like sequences associated with human hepatitis. Nature Medicine.

[bib221] Simpson DI, Knight EM, Courtois G, Williams MC, Weinbren MP, Kibukamusoke JW (1967). Congo virus: a hitherto undescribed virus occurring in Africa. I. Human Isolations-Clinical Notes. East African Medical Journal.

[bib222] Smith AW, Berry ES, Skilling DE, Barlough JE, Poet SE, Berke T, Mead J, Matson DO (1998). In vitro isolation and characterization of a calicivirus causing a vesicular disease of the hands and feet. Clinical Infectious Diseases.

[bib223] Smithburn KC, Hughes TP, Burke AW, Paul JH (1940). A Neurotropic Virus Isolated from the Blood of A Native of Uganda 1. The American Journal of Tropical Medicine and Hygiene.

[bib224] Smithburn KC, Mahaffy AF, Paul JH (1941). Bwamba Fever and Its Causative Virus. The American Journal of Tropical Medicine and Hygiene.

[bib225] Smithburn KC, Haddow AJ, Mahaffy AF (1946). A neurotropic virus isolated from Aedes mosquitoes caught in the Semliki forest. The American Journal of Tropical Medicine and Hygiene.

[bib226] Smithburn KC (1952). Neutralizing antibodies against certain recently isolated viruses in the sera of human beings residing in East Africa. Journal of Immunology.

[bib227] Smithburn KC, Kokernot RH, Weinbren MP, De Meillon B (1957). Studies on arthropod-borne viruses of Tongaland. IX. Isolation of Wesselsbron Virus from a Naturally Infected Human Being and from Aedes (Banksinella) Circumluteolus Theo. The South African Journal of Medical Sciences.

[bib228] Smithburn KC, Paterson HE, Heymann CS, Winter PA (1959). An agent related to Uganda S virus from man and mosquitoes in South Africa. South African Medical Journal = Suid-Afrikaanse Tydskrif Vir Geneeskunde.

[bib229] Smuts H, Workman L, Zar HJ (2008). Role of human metapneumovirus, human coronavirus NL63 and human bocavirus in infants and young children with acute wheezing. Journal of Medical Virology.

[bib230] Song G, Qiu XZ, Ni DS, Zhao JN, Kong BX (1982). Etiological studies of epidemic hemorrhagic fever I. Virus Isolation in Apodemus Agrarius from Non-Endemic Area and Its Antigenic Characterization [Article in Chinese. Zhongguo Yi Xue Ke Xue Yuan Xue Bao Acta Academiae Medicinae Sinicae.

[bib231] Sridhar S, Yip CCY, Wu S, Cai J, Zhang AJ-X, Leung K-H, Chung TWH, Chan JFW, Chan W-M, Teng JLL, Au-Yeung RKH, Cheng VCC, Chen H, Lau SKP, Woo PCY, Xia N-S, Lo C-M, Yuen K-Y (2018). Rat Hepatitis E Virus as Cause of Persistent Hepatitis after Liver Transplant. Emerging Infectious Diseases.

[bib232] Stokes A, Bauer JH, Hudson NP (1928). THE TRANSMISSION OF YELLOW FEVER TO MACACUS RHESUS. Journal of the American Medical Association.

[bib233] Stremlau MH, Andersen KG, Folarin OA, Grove JN, Odia I, Ehiane PE, Omoniwa O, Omoregie O, Jiang P-P, Yozwiak NL, Matranga CB, Yang X, Gire SK, Winnicki S, Tariyal R, Schaffner SF, Okokhere PO, Okogbenin S, Akpede GO, Asogun DA, Agbonlahor DE, Walker PJ, Tesh RB, Levin JZ, Garry RF, Sabeti PC, Happi CT (2015). Discovery of novel rhabdoviruses in the blood of healthy individuals from West Africa. PLOS Neglected Tropical Diseases.

[bib234] Suklin SE, Burns KF, Shelton DF, Wallis C (1962). Bat salivary gland virus: infections of man and monkey. Texas Reports on Biology and Medicine.

[bib235] Sun H, Jia FJ, Huang BC (2016). Research progress and epidemic situation of the Zika Virus [Article in Chinese. Chin J Diagnostics (Electronic Edition).

[bib236] Szmuness W, Dienstag JL, Purcell RH, Stevens CE, Wong DC, Ikram H, Bar-Shany S, Beasley RP, Desmyter J, Gaon JA (1977). The prevalence of antibody to hepatitis A antigen in various parts of the world: A pilot study. American Journal of Epidemiology.

[bib237] Tang FF, Wu SY, Huang YT, Wen ZQ (1958). Research on the isolation of measles virus [Article in Chinese. Chinese Science Bulletin.

[bib238] Tang Y, Gao X, Diao Y, Feng Q, Chen H, Liu X, Ge P, Yu C (2013). Tembusu virus in human, China. Transboundary and Emerging Diseases.

[bib239] Tao Z, Wang H, Zhang W, Xu A (2019). Novel astrovirus types circulating in Shandong Province (Eastern China) during 2016: A clinical and environmental surveillance. Journal of Clinical Virology.

[bib240] Taylor RM, Hurlbut HS, Work TH, Kingston JR, Frothingham TE (1955). Sindbis virus: a newly recognized arthropodtransmitted virus. The American Journal of Tropical Medicine and Hygiene.

[bib241] Taylor MB, Schildhauer CI, Parker S, Grabow WO, Xi J, Estes MK, Cubitt WD (1993). Two successive outbreaks of SRSV-associated gastroenteritis in South Africa. Journal of Medical Virology.

[bib242] Taylor-Robinson D (1963). Studies on some viruses (rhinoviruses) isolated from common colds. Archiv Fur Die Gesamte Virusforschung.

[bib243] Taylor-Robinson D, Tyrrell DA (1963). Virus diseases on Tristan da Cunha. Transactions of the Royal Society of Tropical Medicine and Hygiene.

[bib244] Tesh RB, Watts DM, Russell KL, Damodaran C, Calampa C, Cabezas C, Ramirez G, Vasquez B, Hayes CG, Rossi CA, Powers AM, Hice CL, Chandler LJ, Cropp BC, Karabatsos N, Roehrig JT, Gubler DJ (1999). Mayaro virus disease: an emerging mosquito-borne zoonosis in tropical South America. Clinical Infectious Diseases.

[bib245] Tomori O, Fabiyi A, Murphy F (1976). Characterization of Orungo virus, an orbivirus from Uganda and Nigeria. Archives of Virology.

[bib246] Tomori O, Morikawa S, Matsuura Y, Kitamura T (1986). Antibody to Japanese strain of haemorrhagic fever with renal syndrome (HFRS) virus in Nigerian sera. Transactions of the Royal Society of Tropical Medicine and Hygiene.

[bib247] Venter M, Lassaunière R, Kresfelder TL, Westerberg Y, Visser A (2011). Contribution of common and recently described respiratory viruses to annual hospitalizations in children in South Africa. Journal of Medical Virology.

[bib248] Virus Research Group of Kun Number 323 Unit, The Chinese People’s Liberation Army (1975). Isolation, identification and serological studies of a coronavirus strain [Article in Chinese. Acta Microbiologica Sinica.

[bib249] Wang WS, Zhao CL (1956). Isolation and identification of forest encephalitis virus [Article in Chinese. Journal of Harbin Medical University.

[bib250] Wang TJ, Sun WC, Fang Z, Du SM (1958). Etiology of Mumps in Beijing [Article in Chinese. National Medical Journal of China.

[bib251] Wang CA, Hu CW, Huang FL, Chen X, Hung T (1987). A novel discovered rotavirus from adult acute diarrhoeal patients in China [Article in Chinese. Chinese Journal of Virology.

[bib252] Wang Y, Okamoto H, An P, Chen HS, Liu YL, Wang FS (1996). Infection of hepatitis G virus among blood donors in China [Article in Chinese. Journal of Beijing Medical University.

[bib253] Wang J, Zhang H, Fu S, Wang H, Ni D, Nasci R, Tang Q, Liang G (2009). Isolation of kyasanur forest disease virus from febrile patient, yunnan, china. Emerging Infectious Diseases.

[bib254] Wang Y, Li Y, Jin Y, Li D, Li X, Duan Z-J (2013). Recently identified novel human astroviruses in children with diarrhea, China. Emerging Infectious Diseases.

[bib255] Wang H, Wan Z, Xu R, Guan Y, Zhu N, Li J, Xie Z, Lu A, Zhang F, Fu Y, Tang S (2018). A Novel Human Pegivirus, HPgV-2 (HHpgV-1), Is Tightly Associated With Hepatitis C Virus (HCV) Infection and HCV/Human Immunodeficiency Virus Type 1 Coinfection. Clinical Infectious Diseases.

[bib256] Weaver SC (2013). Urbanization and geographic expansion of zoonotic arboviral diseases: mechanisms and potential strategies for prevention. Trends in Microbiology.

[bib257] Webster LT, Fite GL (2009). A Virus Encountered in the Study of Material from Cases of Encephalitis N the St. Louis and Kansas City Epidemics Of.

[bib258] Wen CC, Chu CM (1957). Survey of influenza antibodies in normal human sera in Peking. Chinese Medical Journal (Peking, China).

[bib259] Wilhelm N, Alexis T (1933). Rabies in South Africa: Occurrence and Distribution of Cases During 1932. Onderstepoort Journal of Veterinary Science and Animal Industry.

[bib260] Williams MC, Woodall JP (1961). O’nyong-nyong fever: An epidemic virus disease in East Africa. Transactions of the Royal Society of Tropical Medicine and Hygiene.

[bib261] Williams MC, Woodaal JP, Corbett PS (1965). Nyando Virus: a Hitherto Undescribed Virus Isolated from Anopheles Funestus Giles Collected in Kenya. Archiv Fur Die Gesamte Virusforschung.

[bib262] Williams CK, Alabi GO, Junaid TA, Saxinger C, Gallo RC, Blayney DW, Blattner WA, Greaves MF (1984). Human T cell leukaemia virus associated lymphoproliferative disease: report of two cases in Nigeria. British Medical Journal (Clinical Research Ed.).

[bib263] Wolfaardt M, Taylor MB, Booysen HF, Engelbrecht L, Grabow WO, Jiang X (1997). Incidence of human calicivirus and rotavirus infection in patients with gastroenteritis in South Africa. Journal of Medical Virology.

[bib264] Woo PCY, Lau SKP, Chu C, Chan K, Tsoi H, Huang Y, Wong BHL, Poon RWS, Cai JJ, Luk W, Poon LLM, Wong SSY, Guan Y, Peiris JSM, Yuen K (2005). Characterization and complete genome sequence of a novel coronavirus, coronavirus HKU1, from patients with pneumonia. Journal of Virology.

[bib265] Woolhouse MEJ, Brierley L, McCaffery C, Lycett S (2016). Assessing the Epidemic Potential of RNA and DNA Viruses. Emerging Infectious Diseases.

[bib266] Woolhouse MEJ, Brierley L (2018). Epidemiological characteristics of human-infective RNA viruses. Scientific Data.

[bib267] Work TH (1964). SEROLOGICAL EVIDENCE OF ARBOVIRUS INFECTION IN THE SEMINOLE INDIANS OF SOUTHERN FLORIDA. Science (New York, N.Y.).

[bib268] World Health Organisation (2020). Coronavirus disease (COVID-2019) situation reports. https://www.who.int/emergencies/diseases/novel-coronavirus-2019/situation430.

[bib269] Wu JR, Che JL, Wu GQ, Lin SQ (1960). Investigation on Coxsackie Virus Disease in Fujian Province [Article in Chinese. National Medical Journal of China.

[bib270] Wu BQ (1981). Report of four cases of rabies encephalitis. New Medicine.

[bib271] Xiang Z, Gonzalez R, Xie Z, Xiao Y, Chen L, Li Y, Liu C, Hu Y, Yao Y, Qian S, Geng R, Vernet G, Paranhos-Baccalà G, Shen K, Jin Q, Wang J (2008). Human rhinovirus group C infection in children with lower respiratory tract infection. Emerging Infectious Diseases.

[bib272] Xiao MH, Ye ZZ, Zhang ZL, Tian XQ, Zheng JM, Liu ZY (1985). An epidemic of hand-foot-and-mouth disease due to Coxsackie A16 in Tianjin City [Article in Chinese. Tianjin Medical Journal.

[bib273] Xu AY, Pang QF, Qiu FX (1981). Detection of astrovirus in faeces of infants with gastroenteritis in autumn [Article in Chinese. Journal of Medical Research.

[bib274] Xu Z, Shen FM, Xu ZY, Huang QS (1990a). HCV infection and primary liver cell cancer [Article in Chinese. Tumor (Shanghai).

[bib275] Xu PT, Wang YM, Zuo JM, Lin JW, Xu PM (1990b). New orbiviruses isolated from patients with unknown fever and encephalitis in Yunnan Province [Article in Chinese. Chinese Journal of Virology.

[bib276] Xu CH, Peng YF, Bai ZJ, Tian XD, Lin LH, Chen CH, Fang MY, Jiang LH (1999). Seroepidemiological survey of arbovirus in Hainan Province in 1998 [Article in Chinese. Chinese Journal of Epidemiology.

[bib277] Yan YS, Zheng ZS, Chen G, Zheng J, Yan PP, Shao YM (2000). Confirmation of the first HIV-2 case in China [Article in Chinese. Journal of Chinese AIDS&STD Prevention and Control.

[bib278] Yang JM, Yin GQ, Feng YH, Luo ZY, Jiao JF, Zhang ZQ (1996). Superinfection of colti virus and Japanese encephalitis virus [Article in Chinese. Journal of Nanjing Railway Medical College.

[bib279] Yang S, Zhang W, Shen Q, Yang Z, Zhu J, Cui L, Hua X (2009). Aichi virus strains in children with gastroenteritis, China. Emerging Infectious Diseases.

[bib280] Yang Y, Ju A, Xu X, Cao X, Tao Y (2016). A novel type of cosavirus from children with nonpolio acute flaccid paralysis. Virology Journal.

[bib281] Yen CH (1941). Isolation of a Virus from an Acute Encephalitis Case in Peiping. Experimental Biology and Medicine.

[bib282] Yen CH, Hsü YK (1941). ISOLATION OF A VIRUS FROM A CASE OF ACUTE POLIOMYELITIS IN PEIPING: WITH HISTOPATHOLOGICAL STUDIES. Chinese Medical Journal.

[bib283] Yen YC, Kong LX, Lee L, Zhang YQ, Li F, Cai BJ, Gao SY (1985). Characteristics of Crimean-Congo hemorrhagic fever virus (Xinjiang strain) in China. The American Journal of Tropical Medicine and Hygiene.

[bib284] Yolken R, Dubovi E, Leister F, Reid R, Almeido-Hill J, Santosham M (1989). Infantile gastroenteritis associated with excretion of pestivirus antigens. Lancet (London, England).

[bib285] Yu JQ, Chang RX, He CJ, Guan QH, Xie JP (1987). Serological study of 722 infants with viral pneumonia [Article in Chinese. Guangdong Medical Journal.

[bib286] Zhang Q (1957). Process of isolating influenza virus in 1956 [Article in Chinese. Biological Products Newsletter.

[bib287] Zhang YZ, Zhou DJ, Xiong Y, Chen XP, He YW, Sun Q, Yu B, Li J, Dai YA, Tian JH, Qin XC, Jin D, Cui Z, Luo XL, Li W, Lu S, Wang W, Peng JS, Guo WP, Li MH, Li ZJ, Zhang S, Chen C, Wang Y, de Jong MD, Xu J (2011). Hemorrhagic fever caused by a novel tick-borne Bunyavirus in Huaiyangshan, China. Zhonghua Liu Xing Bing Xue Za Zhi = Zhonghua Liuxingbingxue Zazhi.

[bib288] Zhang F, Chase-Topping M, Guo CG, van Bunnik BAD, Brierley L, Woolhouse MEJ (2020). Global discovery of human-infective RNA viruses: A modelling analysis. PLOS Pathogens.

[bib289] Zhao JM, Qiang BQ, Zhao TX, Song YY, Deng J, Chen XP, Ou JG, Guo YR, Zhao YN, Cheng H, Zhang Q (1995). Detection of diarrhoea viruses in children with acute gastroenteritis [Article in Chinese. Chinese Journal of Experimental and Clinical Virology.

[bib290] Zoll J, Erkens Hulshof S, Lanke K, Verduyn Lunel F, Melchers WJG, Schoondermark-van de Ven E, Roivainen M, Galama JMD, van Kuppeveld FJM (2009). Saffold virus, a human Theiler’s-like cardiovirus, is ubiquitous and causes infection early in life. PLOS Pathogens.

