# Supplementary material for: Predictors of human-infective RNA virus discovery in the United States, China, and Africa, an ecological study
Source: eLife. 2022 Jun 6;11:e72123. doi: 10.7554/eLife.72123 (PMC9278958; doi:10.7554/eLife.72123)
Supplement: Transparent reporting form [file elife-72123-transrepform1.docx]

***eLife’s* transparent reporting form**

We encourage authors to provide detailed information *within their submission* to facilitate the interpretation and replication of experiments. Authors can upload supporting documentation to indicate the use of appropriate reporting guidelines for health-related research (see [EQUATOR Network](http://www.equator-network.org/%20)), life science research (see the [BioSharing Information Resource](https://biosharing.org/)), or the [ARRIVE guidelines](http://www.plosbiology.org/article/info:doi/10.1371/journal.pbio.1000412) for reporting work involving animal research. Where applicable, authors should refer to any relevant reporting standards documents in this form.

If you have any questions, please consult our Journal Policies and/or contact us: [editorial@elifesciences.org](mailto:editorial@elifesciences.org).

**Sample-size estimation**

- You should state whether an appropriate sample size was computed when the study was being designed
- You should state the statistical method of sample size computation and any required assumptions
- If no explicit power analysis was used, you should describe how you decided what sample (replicate) size (number) to use

Please outline where this information can be found within the submission (e.g., sections or figure legends), or explain why this information doesn’t apply to your submission:

The target sample is all human-infective RNA virus species in the United States (95), China (80) and Africa (107). No explicit power analysis was used in this study, because we included all available subjects of virus species in each region.

**Replicates**

- You should report how often each experiment was performed
- You should include a definition of biological versus technical replication
- The data obtained should be provided and sufficient information should be provided to indicate the number of independent biological and/or technical replicates
- If you encountered any outliers, you should describe how these were handled
- Criteria for exclusion/inclusion of data should be clearly stated
- High-throughput sequence data should be uploaded before submission, with a private link for reviewers provided (these are available from both GEO and ArrayExpress)

Please outline where this information can be found within the submission (e.g., sections or figure legends), or explain why this information doesn’t apply to your submission:

## Data collection was performed by two individuals (Feifei Zhang and Chuan-Guo Guo) independently and discrepancies were resolved by discussion with a third individual (Mark E.J. Woolhouse)(see Data sets of human-infective RNA viruses in three regions in Materials and Methods). Codes for data analysis were also validated by a second author (Chuan-Guo Guo) and all attempts at replication were successful (see Author contributions).

**Statistical reporting**

- Statistical analysis methods should be described and justified
- Raw data should be presented in figures whenever informative to do so (typically when N per group is less than 10)
- For each experiment, you should identify the statistical tests used, exact values of N, definitions of center, methods of multiple test correction, and dispersion and precision measures (e.g., mean, median, SD, SEM, confidence intervals; and, for the major substantive results, a measure of effect size (e.g., Pearson's r, Cohen's d)
- Report exact p-values wherever possible alongside the summary statistics and 95% confidence intervals. These should be reported for all key questions and not only when the p-value is less than 0.05.

Please outline where this information can be found within the submission (e.g., sections or figure legends), or explain why this information doesn’t apply to your submission:

The Poisson boosted regression trees (BRT) model we used in this study is described clearly in Materials and Methods. Raw data on human-infective RNA virus species in three regions were shown in Appendix 1**—**table 1 and were visualised in Figures 1-3. For the boxplots in Figure 4 and Appendix 3**—**figure 7 to Appendix 3**—** figure 8, the median, interquartile range, minimum, maximum and outliers were shown. In Appendix 3**—**figure 3 to Appendix 3**—** figure 6 and Appendix 1**—**table 4, the median and 95% quantiles were shown.

We did not provide the exact values of N, because our observational unit is the spatial grid cell and the number of grid cells varies between the 1000 replicate samples. Unlike the traditional, significance-based approaches, BRT assesses the individual effect of each predictor by estimating the relative contribution of each to the predictions, so the exact p values were also not provided.

(For large datasets, or papers with a very large number of statistical tests, you may upload a single table file with tests, Ns, etc., with reference to sections in the manuscript.)

**Group allocation**

- Indicate how samples were allocated into experimental groups (in the case of clinical studies, please specify allocation to treatment method); if randomization was used, please also state if restricted randomization was applied
- Indicate if masking was used during group allocation, data collection and/or data analysis

Please outline where this information can be found within the submission (e.g., sections or figure legends), or explain why this information doesn’t apply to your submission:

As we mentioned earlier, the observational unit in the study is the 1° resolution grid cell (approximately 110 km at the equator). In each region, the covered grid cells for discovery locations of all virus species were assigned to the study group (presence), and areas with no virus discovery were assigned to the control group (absence, see Boosted regression trees modelling in Materials and Methods). In order to minimise the effect of spatial uncertainty of virus discovery data on our modelling, we performed 1000 times bootstrap resampling for those discovery locations reported as polygons. We assumed each grid cell in the polygon has the equal chance to be selected, and for each virus record we selected one grid cell randomly from the polygon for each subsample. A ratio of 1:2 for presence to absence constituted each subsample, i.e., for each grid cell with virus discovery, two grid cells with no discovery were randomly selected from ‘virus discovery free’ areas at all time points within the region.

**Additional data files (“source data”)**

- We encourage you to upload relevant additional data files, such as numerical data that are represented as a graph in a figure, or as a summary table
- Where provided, these should be in the most useful format, and they can be uploaded as “Source data” files linked to a main figure or table
- Include model definition files including the full list of parameters used
- Include code used for data analysis (e.g., R, MatLab)
- Avoid stating that data files are “available upon request”

Please indicate the figures or tables for which source data files have been provided:

Both our data and codes are available via figshare at <https://doi.org/10.6084/m9.figshare.15101979> and were mentioned in Data availability statement.
